# Epigenetic control of type III interferon expression by 8-oxoguanine and its reader 8-oxoguanine DNA glycosylase1

**DOI:** 10.3389/fimmu.2023.1161160

**Published:** 2023-08-04

**Authors:** Yaoyao Xue, Lang Pan, Spiros Vlahopoulos, Ke Wang, Xu Zheng, Zsolt Radak, Attila Bacsi, Lloyd Tanner, Allan R. Brasier, Xueqing Ba, Istvan Boldogh

**Affiliations:** ^1^Department of Microbiology and Immunology, University of Texas Medical Branch, Galveston, TX, United States; ^2^Key Laboratory of Molecular Epigenetics of Ministry of Education, School of Life Science, Northeast Normal University, Changchun, China; ^3^Horemeio Research Laboratory, First Department of Pediatrics, National and Kapodistrian, University of Athens, Athens, Greece; ^4^Research Institute of Molecular Exercise Science, University of Sport Science, Budapest, Hungary; ^5^Department of Immunology, Faculty of Medicine, University of Debrecen, Debrecen, Hungary; ^6^Respiratory Medicine, Allergology & Palliative Medicine, Lund University and Skåne University Hospital, Lund, Sweden; ^7^Department of Medicine, School of Medicine and Public Health, University of Wisconsin-Madison, Madison, WI, United States

**Keywords:** ROS, NF-κB, IRF, IFN-λ, small airway epithelium, innate immune response

## Abstract

Interferons (IFNs) are secreted cytokines with the ability to activate expression of IFN stimulated genes that increase resistance of cells to virus infections. Activated transcription factors in conjunction with chromatin remodelers induce epigenetic changes that reprogram IFN responses. Unexpectedly, 8-oxoguanine DNA glycosylase1 (Ogg1) knockout mice show enhanced stimuli-driven IFN expression that confers increased resistance to viral and bacterial infections and allergen challenges. Here, we tested the hypothesis that the DNA repair protein OGG1 recognizes 8-oxoguanine (8-oxoGua) in promoters modulating IFN expression. We found that functional inhibition, genetic ablation, and inactivation by post-translational modification of OGG1 significantly augment IFN-λ expression in epithelial cells infected by human respiratory syncytial virus (RSV). Mechanistically, OGG1 bound to 8-oxoGua in proximity to interferon response elements, which inhibits the IRF3/IRF7 and NF-κB/RelA DNA occupancy, while promoting the suppressor NF-κB1/p50-p50 homodimer binding to the IFN-λ2/3 promoter. In a mouse model of bronchiolitis induced by RSV infection, functional ablation of OGG1 by a small molecule inhibitor (TH5487) enhances IFN-λ production, decreases immunopathology, neutrophilia, and confers antiviral protection. These findings suggest that the ROS-generated epigenetic mark 8-oxoGua via its reader OGG1 serves as a homeostatic thresholding factor in IFN-λ expression. Pharmaceutical targeting of OGG1 activity may have clinical utility in modulating antiviral response.

## 1 Introduction

Many of the cellular effects of reactive oxygen species (ROS) are due to oxidation of the base composition of DNA. Among the DNA bases, the most susceptible base to oxidation is guanine (Gua). The generated 8-oxo-7,8-dihydroguanine (8-oxoGua) is a marker of oxidative stress intensity (1). 8-oxoGua is repaired through the evolutionally conserved base excision repair (BER) pathway (2), which is primarily initiated by 8-oxoguanine DNA glycosylase/lyase 1 (OGG1) (3). Recent genome-wide strategies have mapped 8-oxoGua accumulation preferentially within G:C-rich gene regulatory regions, like promoters and super-enhancers (4–6). These studies identified 8-oxoGua as an epigenetic-like mark rather than a mutagenic lesion in shaping gene regulation. Studies have reported that post-translationally modified OGG1 with or without enzymatic activity recognizes 8-oxoGua within and/or in close proximity to *cis* elements of transcription factor (TF) binding sites, like the NF-κB site, facilitating NF-κB binding to inflammatory gene promoters (7–9). Administration of the small molecule TH5487, which inhibits OGG1 recognition of 8-oxoGua, decreases pathophysiological consequences of infections and ameliorates the associated tissue injury (10–12).

Ogg1 knockout in mice or OGG1 depletion by siRNA in human cells lowered ROS and IL-4 levels but increased interferon (IFN) production following challenge with the potent allergen house dust mite extracts (13). Functional inactivation of OGG1 by the specific inhibitor SU0268 induces the release of type I IFNs (triggered by oxidatively damaged DNA via cGAS-STING-IRF3-IFN axis), which decreased *Pseudomonas aeruginosa* loads and halted progression of lung inflammation (14). Inhibition of OGG1 substrate binding by the active site inhibitor TH5487 led to increased expression of type I and type III IFNs, in RSV-infected lungs (15) and in primary alveolar macrophages infected by African swine fever virus (16).

Type III interferons (IFN-λ), consist of IFN-λ1 (IL-29), IFN-λ2, IFN-λ3 (IL-28A, IL-28B), and IFN-λ4 are primarily expressed in mucosal epithelial cells in response to microbial infections (e.g., viruses, bacteria). The detection of pathogen-associated molecular patterns (PAMPs) such as double stranded RNA, which is generated during the life cycle of virus replication, is sensed by the RIG-I like receptors (RLRs). This family includes retinoic acid-inducible gene I (RIG-I) and melanoma differentiation-associated gene 5 (MDA5). Activation of these receptors leads to the recruitment of the mitochondrial antiviral signaling protein, MAVS to mitochondrial associated membranes and peroxisomes. These signaling pathways lead to activation of the transcription factors NF-κB and interferon regulatory factors (IRFs) and expression of both type I and III IFN genes (17). ROS are also involved the expression of IFNs through protein kinase A-driven phosphorylation of NF-κB/RelA and via activation of the RIG-I-IFN pathway (18, Kim, 19–21). The released IFN-λ binds to the IFN-λ receptor, which consists of the IL28 receptor α chain and IL-10 receptor β chain to induce expression of IFN-stimulated genes (ISG). ISGs function to stop the spread of viruses in the host and are crucial in establishing the so called “antiviral state” in neighboring non-infected cells (22).

Here, we tested the hypothesis that the oxidatively-generated epigenetic mark 8-oxoGua and its reader OGG1 controls expression of epithelial type III IFNs in RSV-infected human small airway epithelial cells (hSAECs) and in the lungs of infected mice. We show that inhibition of OGG1 binding and genetic ablation of OGG1 leads to significant increases in mRNA and protein levels of IFN-λ. OGG1 controls IFN-λ expression through increasing DNA occupancy of the suppressor NF-κB1/p50-p50 homodimers, and thereby interfering with binding of IRFs and NF-κB/RelA on the IFN-λ gene promoter. Thus, activation of IFN-λ expression via inhibition or genetic ablation of OGG1 ultimately results in a boost in host antiviral and anti-inflammatory responses. This study advances our understanding of how OGG1 regulates host innate immune responses during pulmonary viral infections and demonstrates that modulation of OGG1 activity may be a druggable target with clinical utility.

## 2 Methods and materials

### 2.1 Cell culture and treatment

HEp-2 cell line (ATCC, CCL-23) was grown in Eagle’s minimum essential medium (MEM) containing 10% (v/v) fetal bovine serum (FBS). A549 cell line (ATCC, CCL-185) was grown in Dulbecco’s Modified Eagle’s Medium/Nutrient Mixture F-12 Ham (DMEM/F12) supplemented with 10% (v/v) FBS. FBS (HyClone, SH30084) was obtained from GE Healthcare Life Sciences. All media were supplemented with penicillin (100 units/mL; Gibco, Life Technologies, Inc.) and streptomycin (100 µg/mL; Gibco, Life Technologies, Inc.). Human small airway epithelial cell line (hSAEC) was cultured in small airway epithelial growth media (Promo Cell, C-21070), supplemented with supplement Mix (Promo Cell, C-39175) containing 0.004 µg/mL Bovine Pituitary Extract, 10 ng/mL Epidermal Growth Factor (recombinant human), 5 µg/mL Insulin (recombinant human), 0.5 µg/mL Hydrocortisone, 0.5 µg/mL Epinephrine, 6.7 ng/mL Triiodo-L-thyronine, 10 µg/mL Transferrin (recombinant human), 0.1 ng/mL Retinoic Acid, 2.5 mg/mL, Bovine Serum Albumin-Fatty Acid Free (BSA-FAF).

We confirmed that TH5487 (10 µM) inhibits RSV-induced cytokine production without inducing cell death at this concentration as measured by lactate dehydrogenase assay (10, 15). TH2840 (inactive analog of TH5487), or O8 (Sigma, SML1697) were added into culture media at 10 μM concentration 1 h prior and after RSV infection every 8 h. (αR)-α-[[(1,2-Dihydro-2-oxo-6-quinolinyl) sulfonyl] amino]-N-(2-furanylmethyl)-2-methoxy-N-(2-thienylmethyl)-benzeneacetamide (OSMI 1, Sigma-Aldrich, SML1621) was used at a concentration of 1 mM. OGG1 knockout was performed using CRISPR/Cas9 technology as described previously (10). Briefly, targeting sequences of OGG1 5′-GATGCGGGCGATGTTGTTGTTGG-3′ and 5′- AACAACATCGCCCGCATCACTGG-3′ were introduced into pSpCas9(BB)-2A-Puro expression vector. Following lipofectamine 2000 transfection (ThermoFisher Sci/Invitrogen, 11668027) into hSAECs, 3 µg/mL of puromycin (ThermoFisher, A1113802) was added. Cells were sub-cultured into 24-well plates, and clones were established. OGG1 knockout cultures were maintained in hSAECs growth medium, containing Growth Medium Supplement Mix in the presence of 2 µg/mL puromycin.

### 2.2 Animals

Eight-week-old female and male BALB/c mice (The Jackson Laboratory, Bar Harbor, ME, USA) housed in pathogen-free conditions in the animal research facility of the UTMB (Galveston, Texas) were used for these studies. Randomly selected groups of mice (50% ♂ and 50% ♀) were challenged intranasally (i.n.) with RSV (10^6^ PFU) in 60 μL of pH-balanced saline solution (pH: 7.4) under mild anesthesia (23). RSV viral titers in the lungs were determined by plaque assay. Vehicle or TH5487 (30 mg/kg, in a 100 μL volume of solvent containing 5% DMSO, 10% Tween 80 in saline) was administered via the intraperitoneal (i.p.) route. In some experiments, mice were treated with recombinant IFN-λ2 protein (0.1 mg/kg, PeproTech, 250-33) or anti-IL-28A/IFN-lambda 2 (2.5 mg/kg, R&D Systems, MAB4635) intranasally.

Euthsaia of mice was performed by carbon dioxide (CO_2_) inhalation. Bronchoalveolar lavage fluid (BALF) samples were obtained by infusing 0.7 mL of PBS into the lungs via the trachea, followed by aspiration into a syringe. BALF samples were centrifuged at 500×*g* for 5 min at 4°C, and the resulting supernatants were snap-frozen and stored at ─80°C for further analysis. All experiments were performed according to the NIH Guide for Care and Use of Experimental Animals and approved by the University of Texas Medical Branch (UTMB) Animal Care and Use Committee (approval no. 0807044D).

### 2.3 siRNA depletion of gene expression

Triplicate cultures of hSAECs or A549 cells were transfected with siRNA targeting hOGG1 (Dharmacon, L-005147-00-0020), hMTH1 (Dharmacon, L-005218-00-0020), hNEIL1 (Dharmacon, L-008327-00-0020). The human NF-κB1/p50 siRNA (16708) and human NF-κB/RelA siRNA (AM16708) were purchased from ThermoFisher Scientific. siRNA transfection was performed using Lipofectamine RNAiMAX Transfection Reagent (13778150, ThermoFisher Scientific) according to the manufacturer’s instructions. In controls, non-targeting siRNAs were used. Depleted cells in fresh culture medium were infected with RSV (MOI = 3) for 24h.

### 2.4 Respiratory syncytial virus infection

The human RSV A2 strain (ATCC VR-1544) was propagated using HEp-2 cells (ATCC CCL-23) and then locally purified on discontinuous sucrose gradients as described previously (24, 25). Aliquots of sucrose purified (cytokine and lipopolysaccharide free) RSV virion suspensions were stored at −80°C. For experiments, cell monolayers (80 to 90% confluence) were infected with RSV at the pre-calculated multiplicity of infection (MOI = 3). After 1 h adsorption, the cell monolayer was washed, and culture medium containing 2% FBS was added.

### 2.5 Assessment of intracellular ROS levels

Amplex red assays were carried out as described previously (26, 27) with minor modification. Briefly, RSV-infected hSAECs were incubated for various lengths of time and equal numbers of cells were washed with PBS (pH 7.4), harvested and sonicated (3 × 30 sec) in a reaction buffer containing the Amplex UltraRed. Mixtures were incubated for 5 min; cell debris was removed (1 min 13,500 g) and changes in resorufin fluorescence were determined at 560 and 620 nm (excitation and emission) by using a Synergy H1 Hybrid Multi-Mode Reader (BioTek). To establish the standard curve, increasing concentrations of H_2_O_2_ (0 to 1000 nM) were used. Resorufin formation in cell extracts was inhibited by addition of catalase (5 U/mL, Sigma–Aldrich).

Intracellular ROS levels were also determined by using the fluorogenic probe 5- (and-6)-chloromethyl-2’7’-dichlorodihydrofluorescein diacetate acetyl ester (CM-H_2_DCFDA; C6827 Invitrogen, Eugene, OR) (15). In brief, hSAEC cells were mock or polyinosinic-polycytidylic acid [poly(I:C)] (Sigma-Aldrich, P1530) exposed ± phenyl-alpha-tert-butyl nitrone (100 µM, Sigma-Aldrich, B7263) and loaded with 10 μM CM-H_2_DCF-DA at 37°C for 10 minutes. Cells were then washed with PBS and lysed (50 mM Tris-HCl, pH 7.5, 150 mM NaCl, 1 mM EDTA, 1 mM EGTA, 1% NP-40), then clarified by centrifugation. Changes in DCF fluorescence in supernatant fluids were determined by using a Synergy H1 Hybrid Multi-Mode Reader (BioTek) with excitation/emission at 485 nm/535 nm. Results are expressed as changes in fluorescence units (FU).

### 2.6 Enzyme-linked immunosorbent assay

Commercially available ELISA kits were used to quantify IFN-β (R&D Systems, DY8234-05), IFN-λ2/3 (R&D Systems, DY1789B-05), IFN-γ (BioLegend, 430804), Myeloperoxidase (R&D Systems, DY3667) and Neutrophil Elastase/ELA2 (R&D Systems, DY4517-05) in BALF following the manufacturer’s protocol.

### 2.7 RNA extraction and qRT-PCR

Total RNAs were extracted using a RNeasy Mini kit (Qiagen, 74106) according to the manufacturer’s instructions. Crude RNAs were DNaseI-treated and loaded onto a RNeasy column and subjected to washes with RW1 and RPE buffers. The RNA concentration was determined spectrophotometrically on an Epoch Take-3™ system (Biotek, Winooski, VT) using Gen5 v2.01 software. The quality of the total RNA was confirmed via the 260/280 nm ratio, which varied from 1.9 to 2.0. 500 ng total RNA was used to generate cDNA using iScript reverse transcription supermix (Bio Rad, 1708840). qPCR was performed using specific primers, and cellular GAPDH as an internal control (sequences of primer are listed in **Supplementary Table 1**). Changes in mRNA levels were calculated using the 2-ΔΔCt method.

### 2.8 mRNA stability analyses

To determine decay of endogenous IFN-λ2/3 mRNAs, OGG1 proficient, OGG1 KO cells or hSEACs with functionally (TH5487) inactivated OGG1 were poly(I:C) treated for 30 min and monolayers were washed with PBS. Four hours later 20 µg/mL 5,6-Dichloro-1-β-D-ribofuranosylbenzimidazole (DRB; D1916; Millipore-Sigma, Saint Louis, Missouri, USA) was added. Total RNAs were isolated at DRB addition (0 h), 2, 4, 8 and 18 h using a RNeasy Mini kit (Qiagen) according to the manufacturer’s instructions. Total crude RNAs were DNaseI-treated and loaded onto a RNeasy column and subjected to washes. RNAs eluted with the RNase-free water included in the kit. The RNA concentration was determined spectrophotometrically on an Epoch Take-3™ system (Biotek, Winooski, VT) using Gen5 v2.01 software. The quality of the total RNA was confirmed via the 260/280 nm ratio, which varied from 1.9 – 2.0. 0.5 µg RNA was used to generate cDNA with oligo-dT (Takara, RR037A). The quantities IFNL2/3 mRNAs at each time points were determined by qPCR by normalizing to 18S rRNA. The relative amount of *IFN-λ2/3* mRNA at time 0 h of DRB addition was set at 100% in each cell type (OGG1 proficient, OGG1 KO cells or hSEACs with functionally (TH5487) inactivated OGG1).

### 2.9 Western blot analysis

Cells were lysed in RIPA buffer (Pierce, 89900 ThermoFisher Scientific) containing Protease and Phosphatase Inhibitor Cocktail (78442, Thermo Fisher Scientific), and centrifuged at 15,000 g for 15 min at 4°C. Equal amounts of Proteins were separated by SDS-PAGE electrophoresis, transferred into nitrocellulose membranes, blocked with 5% non-fat dry milk in TBST (20 mM Tris-base, 500 mM NaCl, and 0.05% Tween-20, pH 7.5), incubated overnight at 4°C with primary antibody and subsequently with horseradish peroxidase-conjugated secondary antibody (1:4000 dilution; SouthernBiotech, Birmingham, AL, USA). Blots were visualized using the enhanced chemiluminescence (ECL) detection system (Bio-Rad, 1705061). To identify proteins in DNA-protein complexes, EMSA gels were transferred to PVDF membrane and performed immunoblotting. The following antibodies were used: OGG1 (Abcam, 124741), phospho-IRF3 (D601M) (Cell Signaling, 29047), phospho-IRF7 (Cell Signaling, 5184), NF-κB/p65 (D14E12) XP (Cell Signaling, 8242), NF-κB1 p105/p50 antibody (AF2697 Novus Biochem), β-Tubulin (Santa Cruz, SC-9104) or β-actin (1:500 dilution; sc-1615, Santa Cruz Biotechnology) was used as internal control. Chemiluminescent signals were visualized and quantified by using the Amersham Imager 680 (Global Life Sci. Sol. Marlborough, MA).

### 2.10 Identification of O-GlcNAcylated OGG1

The method for determination of OGG1-specific O-GlcNAcylation was described previously (28) with modifications. Briefly, 500 µg of total protein samples from FLAG-OGG1 expressing hSAECs were subjected to immunoprecipitation (IP) using FLAG antibody or β-actin (1:500 dilution; sc-1615, Santa Cruz Biotechnology) was used as internal control at 4°C overnight. 30 µL of Protein A/G Magnetic beads (Millipore, MAGNA0017) blocked with normal rabbit IgG (Santa Cruz Biotechnology, SC-2027) were added on next day and incubated for 3 h. Immunoprecipitants were washed extensively and analyzed by Western blotting using anti-O-GlcNAc (RL2) (Thermo Fisher Scientific, MAI-072) and anti-FLAG antibodies. Images were acquired with the Amersham Blot and Gel Imager 680 (Global Life Sci. Sol. Marlborough, MA).

### 2.11 Assessment of protein cysteine oxidation

To determine oxidation of OGG1 at cysteine residues cysteine sulfenic acid probe: DCP-Bio1 reagent (NS1226, Millipore Sigma) was used as we described previously (29). Briefly, hSAECs expressing FLAG-tagged OGG1 were RSV-infected (MOI = 3) for increasing lengths of time and lysed in 50 mM Tris, pH 7.5, 150 mM NaCl, 1 mM EDTA, 1 mM EGTA, 1% Nonidet P-40, 2.5 mM sodium pyrophosphate, 1 mM glycerophosphate, 1 mM Na_3_VO_4_, 1 mM NaF, and 20 µg/ml aprotinin/leupeptin/PMSF buffer (pH: 5.5) and 100 µM DCP-Bio1 supplemented with in 200 units/ml Catalase, 100 μM Diethylene triamine penta-acetic acid, and 5 mM iodoacetamide (30, 31). To capture biotin-linked proteins (OGG1)-Cys-OH, lysates were incubated overnight with 20 μL of Streptavidin beads (Thermo Scientific, 11205D). The IPs were washed with lysis buffer and resolved by SDS–PAGE and then subjected to immunoblotting. OGG1 reactions with DCP-Bio1 were detected by enhanced chemiluminescence.

### 2.12 Chromatin immunoprecipitation assay

ChIP experiments were performed using the ChIP-IT Express Kit (53008, Active Motif, CA, USA). Briefly, 1×10^7^ cells were cross-linked in 1% formaldehyde for 10 min followed by mixing with 1×Glycine for 5 min. After washing with chilled PBS, the cells were re-suspended in 300 µL of 1 x lysis buffer containing iron chelator desferioxamine (DFO, Millipore Sigma, 252750). DNA was sheared by sonication and chromatin was incubated with specific antibodies and isotype control IgG overnight at 4°C and collected using Protein A/G Magnetic beads (Millipore, MAGNA0017) for 3 h. ChIP quality antibodies were phospho-IRF3 (D601M, Cell Signaling, 29047), phospho-IRF7 (Cell Signaling, 5184), NF-κB/p65 (D14E12) XP (Cell Signaling 8242S), NF-κB1 p105/p50 antibody (Cell Signaling 3035). Magnetic beads were washed and the immunoprecipitated DNA was eluted in elution buffer (1% SDS and 100 mM NaHCO_3_) at 65°C for 2 h. The precipitated DNA was phenol/chloroform-extracted, precipitated with 100% ethanol, and dried. qRT-PCR reactions were performed in triplicate using SYBR Green PCR Master Mix (Bio-Rad, 1725120) in a CFX 96 real-time PCR detection system (Bio Rad). Primer sequences are listed in **Supplementary Table 2**. ChIP-qPCR calculations were performed as described previously (32). In brief, protein specific Ab ChIP-ed DNA signal strength value was divided by the intensity value of the IgG-ChIP-ed signal, representing the fold enrichment of the protein on the specific region of genomic DNA.

### 2.13 Electrophoretic mobility shift assay

Whole cell lysates (WCL) or nuclear extracts (NE) were prepared using buffer (Cell Signaling, 9803) and protein concentrations were quantified by a Pierce BCA Protein Assay Kit (Thermo Scientific, 23225). For each reaction, 450 fmol 5’-Cy5-labeled probe was incubated with WCL (or NE) in a total volume of 15 µL containing 10 mM Tris-HCl (pH 7.5), 1 mM EDTA, 50 mM NaCl, 2 mM DTT, 5% glycerol, 0.5% NP-40, 10 µg/mL of BSA, 62.5 µg/mL of poly(I:C) for 20 min at room temperature. For recombinant His-IRF3, 1 pmol 5’-Cy5-labeled probe was incubated with increasing concentrations (0, 5, 10 pmol) of His-IRF3 in a total volume of 10 μL containing 10 mM Tris, pH 7.5, 50 mM KCl, 1 mM DTT, 1% glycerol, 5 mM MgCl_2_, 0.05% NP-40, 50 ng/µL poly(I:C) for 30 min at room temperature. For competition assays, increasing concentrations (0, 2, 4, 8 pmol) of OGG1 (ProSpec, ENZ-253) were incubated with probe for 10 min, then WCL or His-IRF3 was added for 20 min at room temperature. Protein-DNA complexes were resolved on a 6% DNA retardation gel (Invitrogen, EC6365BOX) in 0.5 × TBE buffer (100 V for 2h) and visualized using the Amersham Imager 680 (Global Life Sci. Sol. Marlborough, MA). Oligonucleotide sequences from the *IFN-λ2/3* promoter containing consensus binding sites of IRF3 are shown in **Table 1**. Band intensities were quantified using the Image J v1.51 (U. S. NIH, Bethesda, Maryland, USA). In selected experiments DNA-protein complexes were transferred to PVDF membranes and performed immunoblotting using specific antibodies to RelA/p65, NF-κB1/p50, IRF and OGG1 (for antibody specifications, see 2.9. Western blot analysis). Membrane processing was performed as above and Chemiluminescent signals were visualized and quantified using the Amersham Imager 680 (Global Life Sci. Sol. Marlborough, MA).

**Table 1:** Sequences of duplex oligonucleotides from IFN-λ promoter used for electromobility gel shift assay.

**Pr0**: 5’-CTGTGT**TTTCACTTTTC** CTACATCAGCT***GGGACTGCCC*** TTCTGTCAGGGATAA-3’ Cy5-3’-GACACAAAAGTGAAAAGGATGTAGTCGACCCTGAC**GGG** AAGACAGTCCCTATT-5’
**Pr1**: 5’-CTGTGT**TTTCACTTTTC** CTACATCAGCT/G*/ ***GGACTGCCC*** TTCTGTCAGGGATAA-3’ Cy5-3’-GACACAAAAGTGAAAAGGAGTGTAGTCGACCCTGAC**GGG** AAGACAGTCCCTATT-5’	
**Pr1a**: 5’-CTGTGT**TTTCACTTTTC** CTACATCAGCT***GGGACTGCCC*** TTCTGTCAGGGATAA-3’ Cy5-3’-GTCACAAAA/G*/TGAAAAGGATGAGTCGACCCTGAC**GGG** AAGTCAGTCCCTATT-5’
**Pr2**: 5’-CTGTGT**TTTCACTTTTC** CTACATCAGCT***GGGACTGCCC*** TTCTGTCAG/G*/GATAA-3’ Cy5-3’-GACACAAAAGTGAAAAGGATGTGTCGACCCTGAC**GGG** AAGACAGTCCCTATT-5’
**Pr3:** 5’-CTGTGT**TTTCACTTTTC** CTACATCAGCT***TGGACTGCCC*** TTCTGTCA**TGG**ATAA-3’ Cy5-3’-GACACAAAAGTGAAAAGGATGTAGTCGACCCTGAC/G*/ **GG** AAGACAGTCCCTATT-5’
**Pr4:** 5’-CTGTGT**TTTCACTTTTC** CTACATCAGCT***TGGACTGCCC*** TTCTGTCA**T**GGATAA-3’ Cy5-3’-GACACAAAAGTGAAAAGGATGTAGTCGACCCTGAC**TGG**AAGACAGTCACTATT-3’

The consensus binding site of IRF3 is underlined. NF-κB/p50-p65 binding site is italic and underlined. The 8-oxoGua modification is represented as G* in red. In selected Gua runs (bold), G was mutagenized using thymine (T).

### 2.14 Gelatin zymography

BALF from five mice were pooled, treated with protease inhibitors, and homogenized. Protein concentrations were quantified by Pierce BCA Protein Assay Kit. 2 µg of proteins were mixed with non-reducing sample buffer (final concentrations: 0.8% SDS, 5% glycerol, 0.002% bromophenol blue, 25 mM Tris-HCl, pH 6.8) applied directly to a 7.5% acrylamide gel containing gelatin. The gel was run at 150 V until good band separation was achieved. Then the gel was incubated twice with washing buffer (2.5% Triton X-100, 50 mM Tris-HCl, pH 7.5, 5 mM CaCl_2_, 1 µM ZnCl_2_) for 30 min at room temperature to remove SDS from the gel, and rinsed for 5–10 min in incubation buffer (1% Triton X-100, 50 mM Tris-HCl, pH 7.5, 5 mM CaCl_2_, 1 µM ZnCl_2_) at 37°C. Incubation buffer was replaced, and the gel was incubated for another 24 h at 37°C. Subsequently, the gel was stained with staining solution (0.5% Coomassie Brilliant Blue R250 in 40% methanol and 10% acetic acid) for 30 min to 1 h and was then incubated with de-staining solution (40% methanol and 10% acetic acid) until bands were clearly resolved. The activities of MMPs were detected as clear bands against a blue background.

### 2.15 Statistical analysis

Bars represent means ± SD. Results were analyzed for significant differences using unpaired, two-tailed Student’s t-tests. Differences were considered significant at p<0.05.

## 3 Results

### 3.1 Loss of OGG1 function increases *IFN-λ2/3* gene expression

**Figure 1:**
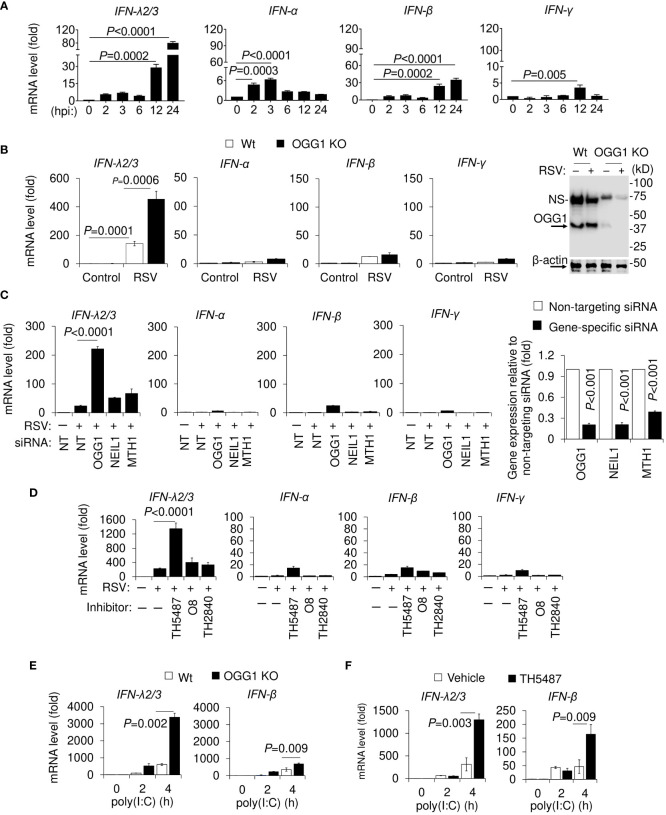
Time course analysis of RSV-induced expression of IFNs in cultured cells and effect of OGG1 knockdown. **(A)** Kinetic changes in mRNA levels of *IFN-λ2/3*, *IFN-α*, *IFN-β* and *IFN-γ* in hSAECs after RSV infection. **(B)** Expression levels of mRNA for *IFN-λ2/3*, *IFN-α*, *IFN-β*, and *IFN-γ* in Wt and OGG1 KO hSAECs, following RSV infection for 24h using an MOI of 3. Most right panel, OGG1 level in KO hSAECs. **(C)** OGG1, but not NEIL1 or MTH1 depletion by targeting siRNA affect *IFN-λ2/3* levels. Far right panel in C shows the mRNA levels of OGG1, NEIL1 and MTH1 after siRNA targeted depletion. **(D)** mRNA levels of IFNs after RSV infection in OGG1 inhibitor-treated hSAECs. **(E, F)** Effect of genetic ablation **(E)** and pharmacological OGG1 inhibition **(F)** on mRNA levels of *IFN-λ2/3* and *IFN-β* after poly(I:C) (100 µg/mL) treatment of cells. Bars represent means ± SD. Statistical analysis, Student’s t-tests (unpaired). NEIL1, Nei Like DNA Glycosylase 1; MTH1, MutY homolog1 or 7,8-dihydro-8-oxoguanine triphosphatase.

To examine the role of OGG1, hSAECs were RSV-infected (MOI = 3) and IFNs expression was determined as a function of time. Results show a ~6-fold increase in *IFN-λ* mRNA levels between 2 and 3 hours post infection (hpi), which then reached over 100-fold by 24 hpi (**Figure 1A**). There was a transient increase in *IFN-α* mRNA levels (between 2 and 6h), while expression of *IFN-β* significantly increased from 12 hpi, reaching a ~30-fold elevation by 24 hpi. Low level expression was observed for *IFN-γ* (**Figure 1A**). Although extent of expression is different, similar results were obtained after RSV infection of hSAEC and A549 cells using higher MOI (MOI = 5, **Supplementary Figures 1A, B**).

Upon loss of OGG1 by CRISPR/Cas9-mediated knockout and siRNA, the mRNA level of *IFN-λ2/3* significantly increased (nearly 500-fold) compared to mock-infected cells to 3-times the level seen in OGG1 proficient hSAECs (**Figure 1B**). Compared to OGG1 proficient, in OGG1 knockdown cells RSV infection increased *IFN-α*, *IFN-β* and *IFN-γ* mRNA levels, but the extent is negligible compared to *IFN-λ* (**Figures 1B, C**). Minor enhancement was observed when knocking down Nei-like DNA glycosylase1 (NEIL1) or 8-oxo-2’-deoxyguanosine triphosphatase a MutT homolog 1 (MTH1) compared to non-targeting siRNA transfected controls (**Figure 1C** and **Supplementary Figure 2A**). Inhibition of OGG1 by the small molecule TH5487 (inhibits OGG1 binding to its DNA substrate) increased *IFN-λ2/3* expression after RSV infection (**Figure 1D** and **Supplementary Figure 2B**). Another nontoxic, selective OGG1 inhibitor O8 (33), which inhibits OGG1 lyase activity but not OGG1 substrate binding, had no effect on IFNs gene expression (**Figure 1D**). Similar data were obtained using hSAECs (**Supplementary Figure 2A**), and A549 cells (**Supplementary Figure 2B**) after infecting cells with RSV (MOI = 3). In controls, the inactive analogs of TH5487, TH2840 or O8, did not alter gene expression levels (**Figure 1D**). These data are in line with substrate binding and excision activity of OGG1 ± inhibitors (**Supplementary Figures 3A, B**).

To test if the modulatory effect of OGG1 on IFNs expression is limited only to virus infected cells, hSAECs were treated with poly(I:C), a synthetic dsRNA and a potent IFN inducer (34). Compared with OGG1 proficient hSAECs, poly(I:C) increased *IFN-λ2/3* mRNA levels from ~500 to ~3400 fold in OGG1 KO hSAECs (**Figure 1E**). Inhibition of OGG1 by TH5487, increased *IFN-λ2/3* mRNA levels from 300 ± 97 to 1700 ± 77-fold (**Figure 1F**). In OGG1 KO and TH5487-treated cells, poly(I:C) also induced expression of *IFN-β* to a lesser level compared to *IFN-λ2/3* (**Figures 1E, F**). These data can be explained by poly(I:C)-induced activation of shared signaling pathways and similarity of the promoter sequences between *IFN-λ2/3* and *IFN-β* (35). Introduction of poly(I:C) into cells increased cellular ROS, which were significantly lowered by the antioxidant phenyl-alpha-tert-butyl nitrone (PBN) (**Supplementary Figure 4A**), also shown previously (36). Poly(I:C) transiently increased 8-oxoGua levels and OGG1 binding as shown by ChIP assays (**Supplementary Figure 4B**). PBN significantly increased IFN-λ and IFN-β expression in poly(I:C)-treated cells by preventing generation of 8-oxoGua in the promoter region (**Supplementary Figure 4C**). Moreover, increased IFN-λ mRNA levels were due to the promoter-driven transcriptional regulation, as there was no difference in mRNA half-life among OGG1 proficient and OGG1 KO cells or TH5487-treated hSAECs (**Supplementary Figure 2C**). Collectively, these data suggest that OGG1 by binding to 8-oxoGua in promoter modulates *IFN-λ2/3* expression not only in RSV-infected, but also in poly(I:C) exposed hSAECs.

### 3.2 8-oxoGua enrichment on promoter, but not OGG1 correlates with *IFN-λ2/3* expression

**Figure 2:**
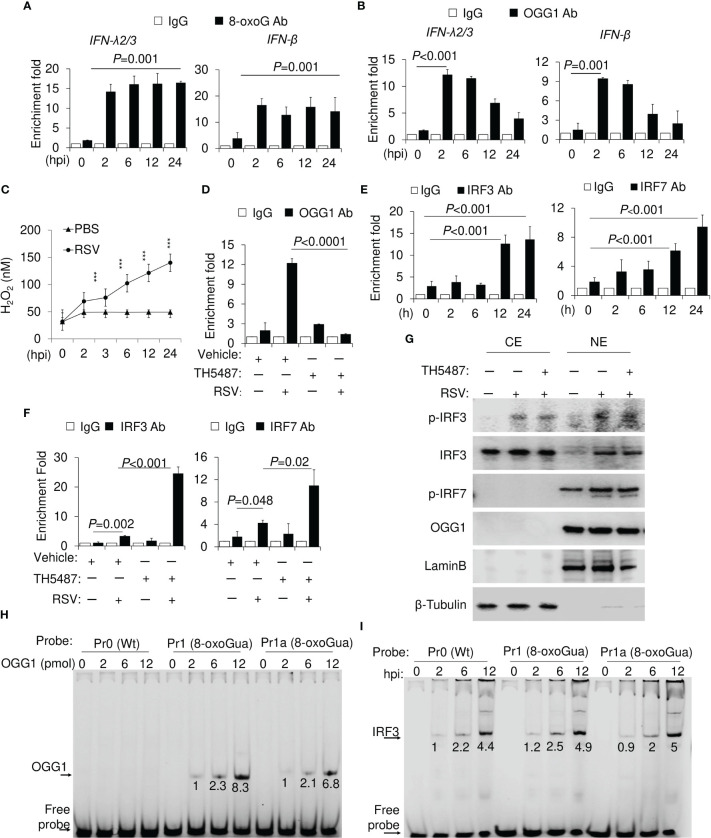
OGG1 and 8-oxoGua enrichment and binding of IRFs to promoters in RSV-infected cells. **(A)** Time course analysis of RSV-induced increase in 8-oxoGua levels in the proximal promoter of *IFN-λ2/3* and *IFN-β*. **(B)** Time course analysis of OGG1 enrichment in *IFN-λ2/3* and *IFN-β* promoter after RSV infection (MOI = 3). **(C)** RSV (MOI = 3) induced ROS levels as assessed by AplexRed assay. ***p < 0.001 **(D)** Effect of the OGG1 inhibitor TH5487 on the OGG1 enrichment on the *IFN-λ* promoter 6 h after RSV infection (MOI = 3). **(E)** Kinetics changes in IRF3 and IRF7 enrichment on IFN-λ2/3 promoter. **(F)** Enrichment of IRF3 and IRF7 on IFN-λ promoter 24 h after RSV infection ± TH5487. **(G)** Protein levels with indicated antibodies shown by western blotting (24 h after RSV infection). **(H)** DNA occupancy of OGG1 on wildtype and 8-oxoGua containing DNA. **(I)** Effect of 8-oxoGua on DNA occupancy of the IRF3 on the IFN-λ promoter. **(A-F)** cross-linked protein-DNA complexes were isolated, and ChIP assays were performed by using Ab to OGG1, 8-oxoGua, IRF3 and IRF7. Fold enrichments were normalized to IgG (Materials and Methods). Bars represent means ± SD. Statistical analysis in **(A-F)** One-way ANOVA (Signal-factor) with Tukey’s multiple comparisons. CE, cytoplasmic extracts. NE, nuclear extracts.

To gain insight into the mechanism by which OGG1 regulates *IFN-λ2/3* repression, we first assessed whether 8-oxoGua, a specific substrate recognized by OGG1, is generated in sequences proximal to the transcription start site (TSS). To address this question, we used chromatin immunoprecipitation (ChIP) assays. As shown in **Figure 2A**, antibody (Ab) to 8-oxoGua extensively ChIP-ed to the TSS adjacent sequences of the *IFN-λ2/3* gene. 8-oxoGua was also increased in the *IFN-β* promoters (**Figure 2A**), at all-time points after RSV infection. As expected, OGG1 enrichment correlated well with the presence of 8-oxoGua in the promoter until 12 hpi, and then decreased on both *IFN-λ2/3* and *IFN-β* promoters at 24 hpi (**Figure 2B**). These data are consistent with increased ROS levels in RSV-infected cells (**Figure 2C**). Binding was specific as OGG1 enrichment can be inhibited by TH5487 (**Figure 2D**). Our data is consistent with previous work where OGG1 is enriched in the proximal promoter regions of *TNF*, *CXCL1* and *IL10* (15).

IRF3 enrichment on the *IFN-λ2/3* promoter was observed after 2 hpi (>3-fold), which continuously increased to 9- and 11-fold by 24 h (**Figure 2E**). Kinetic changes in IRF7 enrichment were similar to IRF3, but its extent is slightly lower (**Figure 2E**, right panel). Both IRF3 and IRF7 abundance on the *IFN-λ2/3* promoter was significantly increased by inhibition of OGG1 (**Figure 2F**). TH5487 has no effect either in IRF3 and IRF7 phosphorylation nor nuclear translocation (**Figure 2G**). To confirm these observations 8-oxoGua was placed in oligos at the 5’ end of Gua runs in proximity of interferon response elements (IREs) (11 bases downstream of IREs, **Table 1**). The 3’ end of oligomers were labelled with Cyanine-5 (Cy5) and EMSAs were performed. Recombinant OGG1 binds only to 8-oxoGua containing DNA in a concentration-dependent manner (**Figure 2H**). Using nuclear extracts (NE) from RSV-infected cells, EMSA analyses confirmed that 8-oxoGua in proximity to IRE (Pr1) or within IREs (Pr1a) did not alter IRF3 binding, and it was similar to that of probe Pr0 (without 8-oxoGua substitution) (**Figure 2I**). These results show that 8-oxoGua does not directly block IRF3 from binding to the *IFN-λ2/3* promoter, rather it is the occupation of OGG1 on the 8-oxoGua containing DNA motifs that blocks IRF3 binding to the *IFN-λ2/3* promoter.

### 3.3 Increased *IFN-λ2/3* expression after inhibition of OGG1 by host-directed O-linked N-glycation

**Figure 3:**
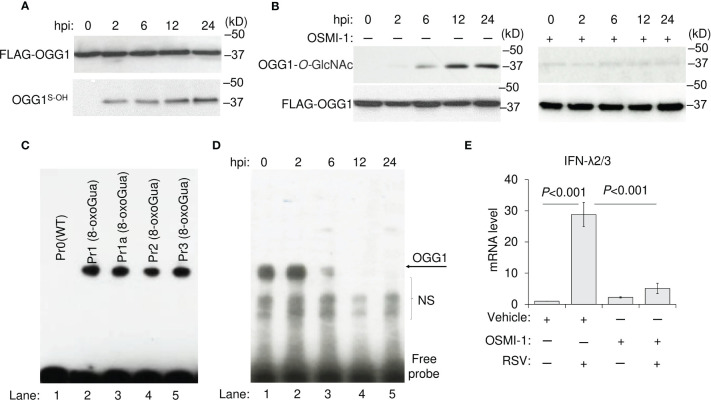
Effect of post-translational modifications of OGG1 on DNA binding and IFN-λ expression in RSV-infected hSAECs. **(A)** Oxidation of OGG1 at cysteine residues in RSV-infected cells as shown by DCP-Biol-1 assays. **(B)** Time course analysis of O-linked N-glycation of OGG1 and its inhibition by the OSMI-1 after RSV infection. **(C)** OGG1 binding in NE from RSV-infected hSAECs to 8-oxoGua-containing (lane 2-5), but not to Wt probes (lane 1). **(D)** OGG1 binding to Pr2 (8-oxoGua) in NE of RSV-infected hSAECs as a function of time. **(E)** IFN-λ2/3 mRNA expression levels in RSV (MOI = 3) infected ± OSMI-1 treated hSAECs as determined by qRT-PCR. In **(A, B)**, FLAG-tagged OGG1 expressing hSAECs cells were RSV infected (MOI = 3) and harvested as indicated, to determine levels of OGG1^S-OH^ and O-linked N-Glycated OGG1 as in Materials and Methods. OGG1^S-OH^: oxidized OGG1 at cysteine residues; NE, nuclear extract; OSMI-1, O-GlcNAc transferase inhibitor. Bars represent means ± SD. Statistical analysis, Student’s t-tests (unpaired).

To obtain insight into the mechanism by which OGG1 inhibition facilitates *IFN-λ2/3* expression, we transgenically expressed FLAG-tagged OGG1 in OGG1-knockout hSAECs, then infected these cells with RSV. The OGG1 level was not affected by RSV infection, but from 2 h onward, OGG1 reacted with DCP-Biol1, indicating oxidation of its cysteine residues (OGG1^S-OH^) (**Figure 3A**). OGG1^S-OH^ binds 8-oxoGua containing oligo with no base excision (**Figure 3C**), as shown previously (7, 9).

In a recent study, we documented activation of the hexosamine biosynthetic pathway and increased activity of O-GlcNAc transferase upon RSV infection (37, 38). Therefore, an antibody (Ab-clone RL2) was utilized against O-linked N-acetylglucosamine that bound strongly to OGG1 from 12 h onwards (**Figure 3B**) and is strong indication for OGG1 inactivation by O-GlcNAcylation (28). Importantly, the inactivation timeline of OGG1 paralleled with the increase in *IFN-λ2/3* mRNA levels, which was shown in **Figure 1A**. The cell-permeable compound, OSMI-1, a specific inhibitor of O-GlcNAc transferase(s), prevents OGG1 O-GlcNAcylation (**Figure 3D**) and decreases *IFN-λ2/3* mRNA levels in RSV-infected cells (**Figure 3E**).

### 3.4 OGG1 does not directly control IRF binding to IRE

ChIP analysis strongly suggests an inverse correlation between OGG1 enrichment and DNA occupancy of IRF3 and 7 on the *IFN-λ2/3* promoter. Next, we examined whether OGG1 directly interferes with IRFs binding to IREs. EMSAs were performed using a probe containing IREs without 8-oxoGua (Pr0), and two probes containing 8-oxoGua, the first (Pr1) of which 8-oxoGua is 11 bases upstream from the IRFs, and the second (Pr2) 8-oxoGua is 29 bases upstream from the IREs. The oligomers used in these experiments were labelled at the 3’-end with Cyanine-5 (Cy5) (**Table 1**).

**Figure 4:**
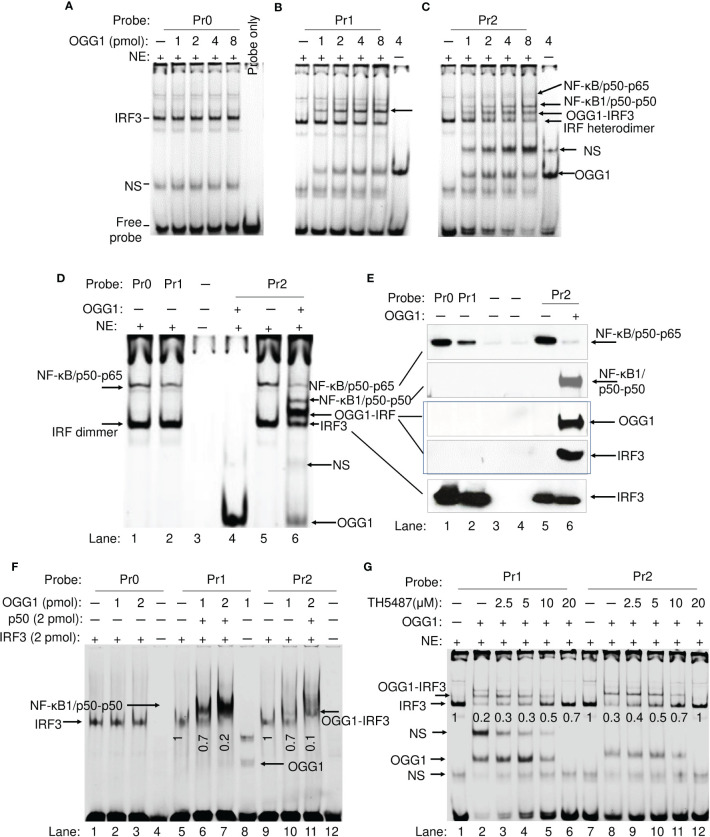
OGG1 indirectly inhibits IRF3 binding to the IRE of the IFN-λ2/3 promoter. **(A)** EMSA shows IRF3 DNA binding from nuclear extract (NE) to wildtype probe (Pr0) ± OGG1. **(B)** EMSA shows IRF3 binding to 8-oxoGua containing Pr1 probe ± OGG1. **(C)** EMSA shows DNA occupancy of IRF3, NFκB1/p50-p50 and NFκB/p50-p65 on 8-oxoGua containing Pr2 probe ± OGG1. **(D)** DNA-protein complexes in NE associated with Pr0, Pr1, and Pr2 ± rOGG1. **(E)** Immunoblot identification of proteins associated with the DNA probe (Pr0, Pr1 and Pr2). **(F)** Reconstruction experiments using OGG1, p50 (2 pmol), and IRF3 (2 pmol), as well as probes without and with 8-oxoGua located 11 and 29 base down-stream from IREs. **(G)** EMSA shows OGG1-IRF-DNA complex is inhibited by TH5487 in a concentration-dependent manner. Sequence of Pr0, Pr1 and Pr2 derived from the *IFN-λ* promoter (**Table 1**).

IRF3 bound efficiently along with NF-κB/p50-p65 to Pr0, and rOGG1 addition had no effect, when using NEs isolated from RSV-infected OGG1 KO hSAECs (**Figures 4A, D**, lane 1). With IRF3 bound to Pr1 containing 8-oxoGua, however, addition of OGG1 resulted in additional distinct complexes containing IRF-OGG1 (**Figure 4B**). Addition of OGG1 into NE, Pr2 resulted in three distinct bands (**Figure 4C, D**, lane 6). OGG1 decreases the IRF-DNA complex in an OGG1 concentration-dependent manner (**Figure 4B, C**). The higher shift indicates the molecular size equals to IRF3-OGG1-DNA complex. A 3rd complex was observed using Pr2, namely NF-κB1/p50-p50-DNA complex, accompanied by a drastic diminution in the DNA shifted by NF-κB/p50-p65 (**Figure 4D**, lane 6). Proteins in EMSA bands were identified as OGG1, IRF3, p50 and RelA/p65 by immunoblotting (**Figure 4E**). These data are in line with the presence of binding sites for IRF, NF-κB and OGG1 in the DNA probe.

In addition to immunoblot analysis, recombinant IRF3, OGG1 and NF-κB1/p50-p50 were used to identify binding characteristics. Addition of IRF3 without OGG1 to Pr0, Pr1 and Pr2 resulted in defined shifts (**Figure 4F**). OGG1 binds only to 8-oxoGua containing probes (**Figure 4F**, lane 8). When OGG1 was added along with IRF3, they formed a higher molecular size complex (**Figure 4F**, lane 6-7 and lane 10 to 11), implying that OGG1 and IRF3 bound to the same DNA. In support of these observations, the OGG1 inhibitor prevented OGG1 binding to DNA and only an IRF3-DNA complex could be seen with highest concentration of TH5487 (**Figure 4G**, densitometry values are under the corresponding bands). Together, these results suggest that OGG1 bound to 8-oxoGua indirectly inhibits expression from *IFN-λ2/3* and additionally, raising the existence of a suppressive protein, namely the NF-κB1/p50-p50 homodimer.

### 3.5 OGG1 8-oxoGua complex facilitates binding of NF-κB1 suppressor to Guanine islets in the *IFN-λ2/3* promoter

**Figure 5:**
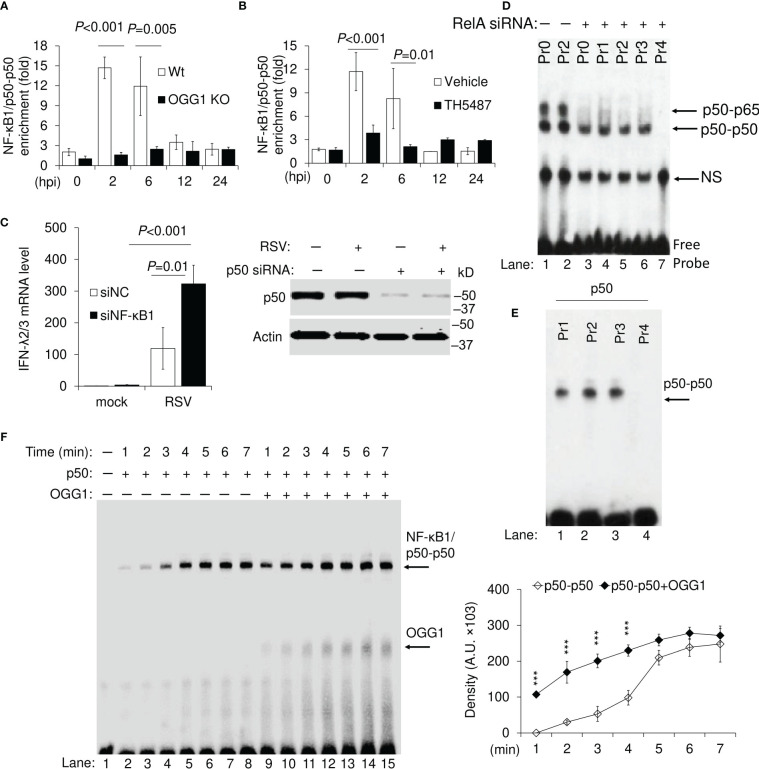
Binding of NF-κB1/p50-p50 suppressor to Gua islets in wildtype and OGG1 knockout RSV-infected cells. **(A)** NF-κB1/p50-p50 enrichment on the IFN-λ2/3 promoter in OGG1 proficient and OGG1 KO cells. **(B)** Effect of OGG1 inhibitor on NF-κB1/p50-p50 enrichment on the *IFN-λ2/3* promoter. **(C)** mRNA levels of IFN-λ2/3 in NF-κB1/p50-p50 proficient and depleted mock and RSV-infected hSAECs. Right panel: Western blot analysis of NF-κB1/p50-p50 in proficient and siRNA-depleted, mock- and RSV-infected hSAECs. **(D)** RelA was depleted by targeted siRNA in hSAECs, and infected with RSV. NE was isolated and incubated with probes to perform EMSA. **(E)** NF-κB1/p50-p50 binding to intact 8-oxoGua containing probes (Pr1,2 and 3), or the mutagenized probe (Pr4). **(F)** Time course analysis of NF-κB1/p50-p50 binding to Pr2 ± OGG1. OGG1 and NF-κB1/p50-p50 proteins were incubated with Pr2 probe for indicated time and subjected to EMSA. Right panel: graphical depiction of band densities. Bars represent means ± SD. Statistical analysis, Student’s t-tests (unpaired). ***p<0.001.

NF-κB1/p50-p50 extensively binds to 8-oxoGua containing oligos, in parallel with a decreased DNA occupancy of IRF3 and NF-κB/p50-p65 in the presence of OGG1 (**Figures 4B-D**). These results imply that OGG1 engaged with 8-oxoGua can allosterically change the adjacent DNA helix, which was documented previously (39, 40). To test this possibility, ChIP assays using Ab to p50 were performed using RSV-infected OGG1 expressing and OGG1 KO cells. Results indicate significant levels of NF-κB1/p50-p50 enrichment on the *IFN-λ2/3* promoter from 2 hpi, which further increased by 6 hpi and then decreased significantly from 6, 12 hpi to near background level by 24 hpi in OGG1 proficient cells (**Figure 5A**). This decrease in NF-κB1/p50-p50 promoter enrichment parallels with the inability of increased O-GlcNAcylated OGG1 to bind the intrahelical DNA substrate (**Figure 3D**). In support of this observation, both in RSV-infected OGG1 KO hSAECs, as well as OGG1 proficient cells treated with TH5487, NF-κB1/p50-p50 enrichment was poor (**Figures 5A, B**). To test whether NF-κB1/p50-p50 is indeed suppressive on *IFN-λ2/3* expression, NF-κB1/p50-p50 was depleted by siRNA in hSAECs (**Figure 5C**, right panel), and cells were infected by RSV for 24 h. ChIP analysis failed to show significant NF-κB1/p50-p50 enrichment on the *IFN-λ2/3* promoter (not shown). In p50 siRNA-treated cells infected with RSV, there was a >300-fold increase in IFN-λ2/3 mRNA expression. This compares to ~100-fold increase in IFN-λ2/3 mRNA expression in non-targeting siRNA transfected cells (**Figure 5C**, left panel). These results suggest an inhibitory role of NF-κB1/p50-p50 on IFN-λ2/3 expression.

Next, we made attempts to identify the sequence requirement for NF-κB1/p50-p50 binding. Results show that OGG1 promotes NF-κB1/p50-p50 DNA occupancy. In both the *IL28A* and *IL28B* promoters, IRF binding sites are in proximity to NF-κB binding elements (5’-**GGG**ACTGCC-3’). There are Gua runs (5’-GGG-3’) upstream and downstream to the NF-κB binding sites, and these binding sites are present on both the sense and antisense DNA strands (**Supplementary Figure 5**). 8-oxoGua was placed individually in the 5’ end of Gua runs (**Table 1**), including NF-κB binding elements as may occur in chromatin due to charge migration (41) and the potential NF-κB1/p50-p50 binding sites including Gua runs were also mutagenized (G→T, Pr3, Pr4). First, NE from nontargeting siRNA transfected RSV-infected from OGG1 expressing hSAECs showed binding of activated NF-κB/p50-p65 heterodimer to ±8-oxoGua probes consistent with NF-κB binding elements (Pr0 and Pr2) (**Figure 5D**, lanes 1-2). NEs from RelA-depleted cells (targeting siRNA to RelA) resulted in binding only of NF-κB1/p50-p50 to probes ±8-oxoGua (**Figure 5D**, lanes 3-4). In the probe where all Gua runs were mutated, no p50-p50 was captured from NEs (**Figure 5D** and 5E, last lanes, Pr4). The EMSA also showed that 8-oxoGua in proximity to the NF-κB site or in Gua runs (Pr1, Pr2, Pr3) had no effect on NF-κB1/p50-p50 binding, but substitution of Gua for T in G-runs (5’-GTG-3’) eliminated the shift of Pr4 (**Figure 5E**, Lane 4). OGG1 binds to 8-oxoGua containing probes, and there is no difference in binding caused by 8-oxoGua being in the sense or in the antisense strand (**Figure 3C**). Results summarized in **Figure 5F** (Pr2, lanes 2-8 and lanes 9-15) clearly suggest that OGG1 increases NF-κB1/p50-p50 binding to 5’-GGG-3’ in a time dependent manner. For example, in the presence of OGG1 at 1 min, levels of DNA-associated NF-κB1/p50-p50 were similar to that of 4 min without OGG1 (compare lane 5 to lane 9, in **Figure 5F**). Binding of NF-κB1/p50-p50 to Gua runs is in line with the strict conservation of the first three guanines in the NF-κB site as determined by structural studies (42).

### 3.6 Pharmacological inhibition of OGG1 increases IFN-λ expression during RSV infection

**Figure 6:**
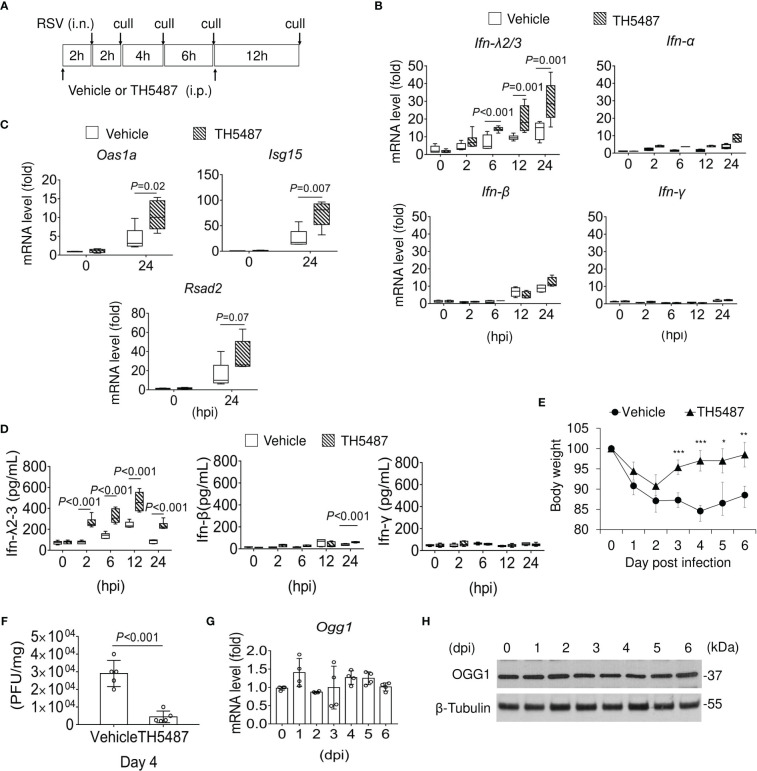
Expression of IFNs in the lungs of mock and RSV-infected mice with or without OGG1 inhibition. **(A)** Infection and treatment schedule of experiment. **(B)** mRNA levels of *Ifn*-λ2/3, *Ifn*-α, *Ifn-β* and *Ifn*-γ in RSV-infected lungs with or without TH5487 treatment. **(C)** mRNA levels of selected IFN-stimulated genes, *Oas1a*, *Isg15* and *Rsad2* in RSV infected lungs ± TH5487. **(D)** IFN-λ, IFN-β and IFN-γ protein levels in BALF as determined by ELISA (n=6). **(E)** Percentage weight loss of mice with and without TH5487 treatment after RSV infection. **(F)** Viral load in lung tissue of vehicle and TH5487 treated RSV infected mice was determined by plaque forming assays on day 4. **(G)** mRNA and **(H)** protein level of OGG1 in the lungs after RSV infection. In **(B-F)** groups of mice were mock or RSV infected (PFU = 10^6^ per lung) and treated i.p with TH5487 (30 mg/kg) or equivalent volume of solvent daily. **(B, C)** mRNA levels were determined by qRT-PCR. Data is shown from three to four independent experiments. PFU, plaque forming unit; BALF, bronchoalveolar lavage fluid; *Oas1a*, 2’-5’-Oligoadenylate Synthase 1; *Isg15*, Interferon-Induced 17-kD/15-kD Protein; *Rsad2*, Radical S-Adenosyl Methionine Domain-Containing Protein 2. Data is expressed as means ± SD. Statistical analysis in B to F, Student’s t-tests (unpaired). *p<0.05, **p<0.01, ***p<0.001.

IFN-λ2/3 has the unique capacity to restrict viral invasion, increase virus clearance and decrease inflammatory responses to preserve the integrity of the mucosal epithelium (43). Therefore, we tested whether OGG1 inhibition altered expression of mucosal IFN-λ2/3 and lung pathology in mice infected with RSV (10^6^ PFU per lung). Vehicle or TH5487 (30 mg/kg) was administered intraperitoneally (i.p.) prior and after infection, as outlined in **Figure 6A**. TH5487 prevented OGG1 binding to substrate-containing DNA in NE both *in vitro* (**Supplementary Figure 6A**) and *in vivo* as measured by ChIP assays (**Supplementary Figure 6B**). 8-oxoGua levels in the same promoter as OGG1 enriched were increased continuously (**Supplementary Figure 6C**). Compared with mock controls, the lungs of RSV-infected mice demonstrated increased mRNA expression of *IFN-λ2/3.* This effect was further enhanced in RSV-infected mice that were treated with TH5487 to inhibit OGG1 (**Figure 6B**). These data support the findings generated with cell culture and *in vitro* assays that showed that OGG1 presence in the promoter inhibits IRF binding via the suppressor NF-κB1/p50-p50 (**Supplementary Figure 6D**, lane 2 and 3). At 24 hpi, OGG1 inactivation by O-GlcNAcylation (**Supplementary Figure 6E**) corelated with increasing binding of IRF3 (**Supplementary Figure 6D**, lane 5 and 6). We note, *Ifn-α* and *Ifn-β* are not significantly increased until 24 hpi in line with accumulation of IFNα, and β expressing immune cells (Natural killer (NK) cells, B- and T-cells and macrophages). *Ifn-γ* expression was not significant during the early phase of RSV infection (**Figure 6B**) as it produced by NK T cells, CD4 Th1 and CD8 cytotoxic T lymphocyte, and effector T cells (44, 45), of which the expression is low even at 24 hpi.

*IFN-λ2/3* expression correlates with a significant induction at the mRNA level of selected interferon stimulated genes (ISG), like Oas1a (2’-5’-Oligoadenylate Synthetase 1), Isg15 (Interferon-stimulated gene 15), and Rsad2 (Radical S-Adenosyl Methionine Domain 2) in the presence of TH5487 at 24 hpi (**Figure 6C**). We examined expression of IFN at protein levels in BALF. Mock- and RSV-infected mice were treated with vehicle or TH5487 (30 mg/kg), and BALF was collected at indicated time post-infection. We showed that treatment with 30 mg/kg TH5487 significantly increased levels of IFN-λ2/3 (**Figure 6D**). Hence, in mice treated with TH5487, IFN-λ2/3 expression in the airways is substantially altered, including a sustained peak response during RSV infection. In addition, we also observed less weight loss in TH5487-treated mice (**Figure 6E**). Furthermore, TH5487-treated mice had lower viral load than vehicle treated mice at day 4 post-infection (**Figure 6F**). These results demonstrate that TH5487 may have a protective role by enhancing host antiviral response upon RSV infection. There were no significant changes in expression of OGG1 mRNA (**Figure 6G**) or protein (**Figure 6H**) 6 days post-RSV infection, indicating that OGG1 function can be targeted at the level of substrate recognition. The decreases in RSV-mediated immune pathology are in line with those observed after IFN-λ addition to animals prior to infection with human metapneumovirus (HMPV) or African swine fever virus (16, 46).

**Figure 7:**
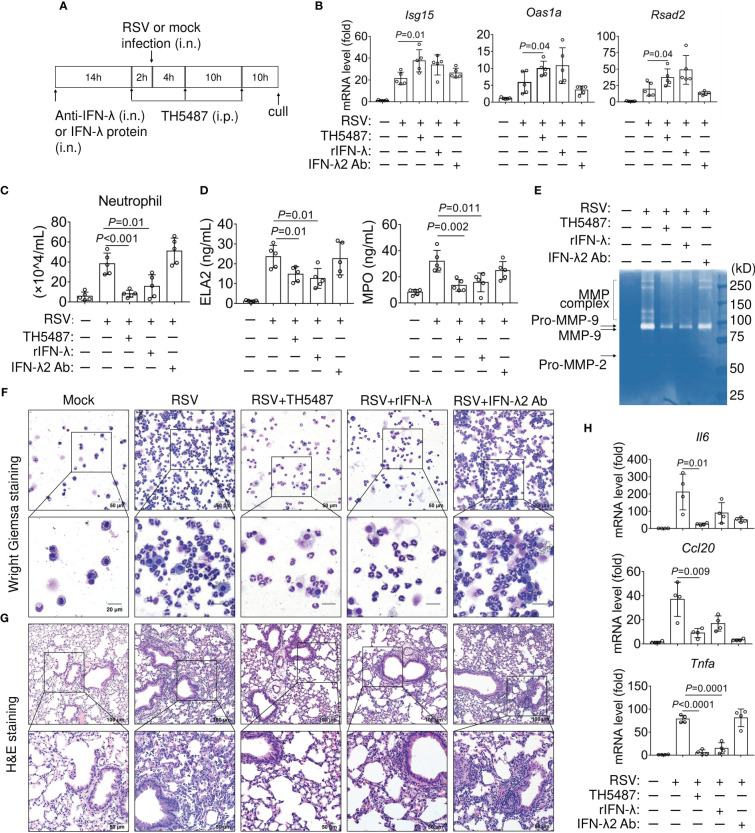
Effect of IFN-λ and TH5487 administration on expression of IFN-stimulated genes and airway inflammation in RSV-infected mice. **(A)** Schematic depiction of experimental design. **(B)** mRNA levels of IFN stimulated genes, *Oas1a*, *Isg15*, and *Rsad2* in the mouse lung as determined by qRT-PCR (n=5). **(C)** Numbers of neutrophils in the BALF. **(D)** Neutrophil Elastase/ELA2 and myeloperoxidase (MPO) in BALF were quantified by ELISA. **(E)** Matrix Metallopeptidase 9 (MMP-9) activity in the BALF from RSV-infected mice ± TH5487, IFN-λ protein or IFN-λ neutralizing antibody was analyzed by gelatin zymography. Pro-MMP-9, MMP-9 are indicated by arrows. **(F)** Representative images of Wright Giemsa-stained cells in BALF from mock- and RSV-challenged mice ± TH5487, IFN-λ protein or IFN-λ neutralizing antibody. **(G)** Representative images of hematoxylin and eosin-stained lung sections after RSV infection ± TH5487, IFN-λ protein or IFN-λ neutralizing antibody. **(H)** mRNA levels of inflammatory cytokines, *Il6*, *Tnfα* and *Ccl20* in the mouse lungs as determined by qRT-PCR. In (B-H) mice were RSV challenged (10^6^ PFU) via the intranasal route, TH5487 (30 mg/kg daily). IFN-λ (0.1 mg per kg), antibody to IFN-λ (2.5 mg per kg) administered i.n. Symbols represent individual mice (n=5). Statistical analysis, One-way ANOVA with Tukey’s multiple comparisons. In (C, D) Statistical analysis, Student’s t-tests (unpaired). *Oas1a*, *Isg15*, and *Rsad2*, as in legend to **Figure 6**; *Il6*, interleukin 6 or interferon beta-2; *Tnfα*, tumor necrosis factor-alpha; *Ccl20*, C-C motif chemokine ligand 20.

The potent immunomodulatory activities of IFN-λ prompted us to further investigate its potential therapeutic activity *in vivo*. First, we blocked early IFN-λ release with anti-IL-28A/IFN-λ (2.5 mg/kg), an IFN-λ neutralizing antibody, during RSV-infection (**Figure 7A**). Then, we administered TH5487 (30 mg/kg) or recombinant IFN-λ2 (rIFN-λ) (0.1 mg/kg) to mock- and RSV-infected mice. Prophylactic treatment with TH5487 and rIFN-λ directly to the upper respiratory tract resulted in increased expression of *Isg15*, *Oas1*a and *Rsad2*. This effect was partially abrogated in mice treated with the neutralizing antibody on day 1 post-infection (**Figure 7B**). Neutrophils respond to cytokines to upregulate antimicrobial functions and exhibit pro-inflammatory activation, which is essential for confronting infection but also induces immunopathology (47). Mouse and human neutrophils both express the type III IFN receptor thus suggesting a possible conserved role for IFN-λ in regulating neutrophil activation. We then tested neutrophil infiltration into BALF of mock- and RSV-infected mice. We found a significant decrease in RSV-induced neutrophils in the presence of TH5487 and rIFN-λ. The neutralizing IFN-λ antibody elicits the highest neutrophil accumulation (**Figure 7C**).

Upon activation, neutrophil elastase (ELA2) and myeloperoxidase (MPO) enhances chromatin de-condensation and promotes formation of neutrophil extracellular traps (NETs) (48). Therefore, we measured levels of ELA2 and MPO in the BALF from mock- and RSV-infected mice. Treatment with TH5487 and rIFN-λ had similar effects in lowering ELA2 and MPO levels in response to RSV infection (**Figure 7D**, left and right panels). In addition to elevated levels of ELA2, the activities of matrix metalloproteinase (MMP) have also been demonstrated in facilitating migration of neutrophils across basement membranes (49). Thus, we performed gelatin zymography to determine MMP activity (50). The degree of digestion of gelatin in BALF samples from RSV-infected mice showed that the proenzyme is converted to an active form, which was hardly seen in mice treated with TH5487 and rIFN-λ, suggesting that these compounds inhibit MMP activity (**Figure 7E**). Additionally, RSV-infected mice treated with anti-IL-28A/IFN-λ (2.5 mg/kg) had a more robust increase in neutrophil infiltration in both BALF (**Figure 7F**) and airway tissues (**Figure 7G**) as compared to TH5487- and rIFN-λ-treated mice. An exacerbated antiviral response is also associated with an increase in pro-inflammatory cytokines. Accordingly, RSV-infected mice demonstrated increased mRNA expression of *Il6*, *Tnfα* and *Ccl20* (**Figure 7H**). In contrast, the expression of these pro-inflammatory cytokines is substantially lower in TH5487- and rIFN-λ-treated mice. IFN-λ2/3 neutralization resulted in the upregulation of *Tnfα* mRNA (**Figure 7H**). Thus, pharmacological inhibition of OGG1 results in a beneficial outcome that is associated with increased IFN-λ2/3 expression. Taken together, these data indicate that OGG1 plays a crucial role in mediating the anti-viral immune response through regulation of IFN-λ2/3.

## 4 Discussion

**Figure 8:**
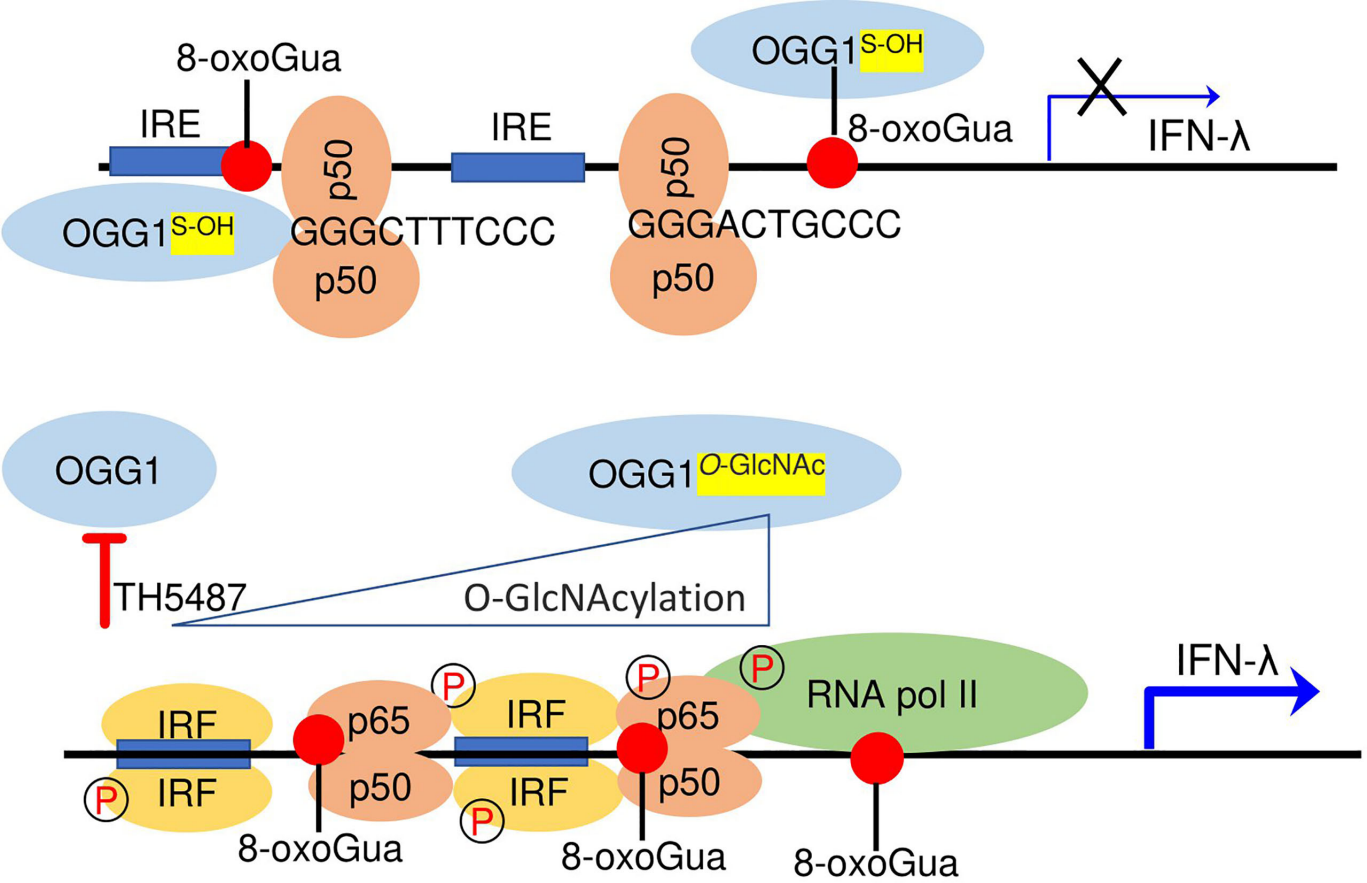
Epigenetic regulation of INF-λ expression in RSV-infected cells. Upper panel, OGG1 interaction with the epigenetic mark 8-oxoGua in promoter facilitates binding of the suppressor NF-κB1/p50-p50 to 5’-GGG-3’ at early time of RSV infection. Lower panel, functional inhibition by TH5487 or O-GlcNAcylation prevent OGG1 to read 8-oxoGua allowing NF-κB/50-p65 and IRF dimer binding for transcriptional activation of IFN-λ in the late-phase of RSV infection.

Herein, we describe an unexpected regulatory circuit controlling IFN-λ expression in RSV-infected hSAECs and mice. Specifically, OGG1 interaction with its substrate inversely correlates with binding of the primary transcription factor(s) IRFs and NF-κB/RelA to IREs for expression of IFN-λs. Mechanistically, allosteric changes in DNA promote binding of the repressor NF-κB1/p50-p50 that decreases DNA occupancy of IRFs and NF-κB/RelA in the chromatin which negatively controls IFN-λ expression. This suppression is reversed by host-driven post-translational modification (O-GlcNAcylation) of OGG1, OGG1 knockdown, and OGG1 inhibition (**Figure 8**). OGG1 knockdown or pharmacological inactivation also increased poly(I:C) induced expression of IFNs, extending the range for OGG1 in controlling the inducible expression of these key immune regulators. We speculate that targeting OGG1 substrates-binding through TH5487 can be utilized to optimize expression of IFNs, thus controlling the extent of innate and adaptive immune responses.

Through specific post-translational modifications, OGG1 binds to covalently modified DNA base 8-oxoGua without excision to facilitate transcription factors binding to DNA motif, mediating timely cellular physiological responses (51). Conventionally, 8-oxoGua is a marker of oxidative stress as Gua has the lowest oxidation potential among nucleobases (52, 53). However, in gene regulatory sequences it can function as an epigenetic-like mark (51, 52, 54, 55) with OGG1 considered a reader. 8-oxoGua can be generated by ROS derived from receptor-driven targeted demethylation of histone H3 lysine 9 at both enhancer and promoter locations by the activation of resident lysine specific demethylase1 (56, 57) and ROS generated by other oxidoreductases (e.g., NADPH oxidases in RSV-infected cells) (58).

In contrast to IFN-λ expression, pro-inflammatory mediators (e.g., TNF, ILs, CCLs, CXCLs) correlated well with the transiently generated epigenetic mark, 8-oxoGua and with enrichment of the reader OGG1 in TSS adjacent promoter regions (9, 15). The TSS-proximal promoter region of cytokines/chemokines and IFN-λ2/3 are guanine-rich, containing Gua islands (e.g., 5’-GGG-3’) and this π-stacking effect becomes more pronounced as the length of the G run increases (52, 59, 60). Therefore, it was not surprising to observe an 8- to 14-fold increase in 8-oxoGua enrichment in the promoters after RSV infection. Its enrichment took place in TSS proximal regions of *IFN-λ* (also *IFN-β*), which both contain a cluster of binding sites for IRFs, and NF-κB/p50-p65. Interestingly, in OGG1-enriched *IFN-λ2/3* promoter, IRFs and NF-κB/RelA enrichment was low or undetectable. However, treatment of RSV-infected hSAECs with the OGG1 inhibitor TH5487 or OGG1 knockdown decreased levels of proinflammatory mediators, while significantly increasing IRFs and NF-κB/p50-p65 promoter enrichment and IFN-λ2/3 expression. These findings imply that OGG1 has a differential effect on gene expression as shown previously (9). In support, ChIP-coupled sequencing studies have shown that stimuli-dependent enrichment of OGG1 occurred over thousands of enhancers and promoters in chromatinized DNA, however, a large portion of OGG1 enrichment was associated with gene activation and another portion with gene silencing (9).

Although an RSV dose added to cells (MOI = 3) activates both IRFs and NF-κB/p50-p65 at early time points, IFN-λ2/3 expression only increased abundantly at 24 hpi. Further investigation showed that there were no changes in OGG1 expression levels, while a post-translational modification by O-GlcNAcylation occurred only at later time points in infected cells. Note that at this time point (24 hpi) expression of selected inflammatory mediators (e.g., TNF, CXCL1) decreased (15), which can now be explained by OGG1 O-GlcNAcylation. This reversible post-translational modification primarily affected serine and threonine residues of OGG1, leading to decreased substrate recognition and damaged base excision (28). OGG1 also showed oxidation at its cysteine residues to sulfenic acid at all time points of infection, like those shown previously (15). Placing these modifications into the context of IFN-λ2/3 expression, OGG1^-S-OH^ can bind to DNA via 8-oxoGua, while O-GlcNAcylated OGG1 poorly recognized its DNA substrate (28). Since the O-GlcNAcylation inhibitor (OSMI-1) suppresses *IFN-λ2/3* expression, it is likely that this decrease is due to the enhanced binding of OGG1 to 8-oxoGua in the *IFN-λ*2/3 promoter. OGG1 O-GlcNAcylation correlated well with increased binding of NF-κB/p50-p65 and IRFs and enhanced IFN-λ2/3 expression. Therefore, in this context, OGG1 inactivation by O-GlcNAc transferase can be considered part of an antiviral response. OGG1 O-GlcNAcylation is in line with RSV-induced increase in uridine 5′-diphosphate-N-acetyl-D-glucosamine (UDP-GlcNAc) levels, a rate-limiting precursor of the O-GlcNAc transferase in the hexosamine biosynthetic pathway shown by our labs and others (37, 38). In addition, NF-κB O-GlcNAcylation robustly increases NF-κB/p65-dependent gene expression (61).

OGG1^S-OH^ can bind 8-oxoGua and interact with the complimentary cytosine, which bends DNA and changes the adjacent DNA architecture. These changes in the DNA landscape are hypothesized to decrease the energy required for transcription factor binding including NF-κB/p50-p65, AP1, SMADs, which results in increased expression from proinflammatory and wound healing genes (9, 36, 39, 51, 53). We note that the DNA mismatch repair pathway permitted survival of influenza virus infected cells that also showed an increase in IFN expression and enabled transcription of host genes in OGG1 proficient cells (62, 63).

From our study it is obvious that OGG1 binding to its epigenetic mark in the *IFN-λ2/3* proximal promoter increases DNA occupancy of the constitutive repressors (e.g., NF-κB1/p50-p50) to Gua island (5’-GGG-3’) within or adjacent to IREs in the *IFN-λ2/3* promoter. The NF-κB1/p50-p50 lacks a transactivation domain such as RelA, c-Rel or RelB and therefore, it has a repressive role on gene expression by interfering with transcription factor binding, thereby decreasing *IFN-λ* expression. Indeed, using wild-type and mutagenized oligo probes, we found that OGG1 increases DNA occupancy of NF-κB1/p50-p50 on Gua runs (5’-GGG-3’). This data can be explained by crystallographic studies showing that p50-p50 makes base-specific contacts with the first three Gua of the NF-κB consensus site (5’-GGGRNYYYCC-3’) (8, 64). In further support, X-ray structures of the NF-κB1/p50-p50 DNA complex show the ability of p50-p50 to efficiently bind to Gua runs (5’-GGG-3’) (42, 65). Specifically, the hydrogen bonds made by His 64 with the N7 group of G5, and by Arg 56 and Arg 54 with both the N7 and O6 groups of G4 and G3 (42, 64). Therefore, NF-κB1/p50-p50 can act as a transcriptional repressor on pro-inflammatory genes (66, 67). Previous studies using molecular, biochemical and structural analyses also showed that the NF-κB1/p50-p50 homodimer significantly decreased expression of a number of IFN-inducible genes via binding to a subclass of guanine-rich IRE sequences (68). Moreover, NF-κB1 knockout mice lacking the p50-p50 have highly increased inflammatory responses to various agents in their lungs, liver, and kidney implying a predominant role for NF-κB1/p50-p50 as a negative regulator of NFκB-driven pro-inflammatory gene expression (69, 70).

The receptor for IFN-λ is primarily expressed on epithelial cells (71) and neutrophils (72), thus suggesting that the protective effects of IFN-λ are prominent specifically in stimulating innate immune cells. Pharmacological inhibition of OGG1 by TH5487 decreased markers of lung injury in RSV-infected mice by IFN-λ-mediated inhibition of RSV replication, neutrophilia, or both. While we have no evidence for direct implication of OGG1 in RSV replication, there was decreased neutrophil numbers, decreased ELA2, MPO and MMP-9 expression. We also observed decreased weight loss and RSV viral titers in RSV-infected lungs. These results are in line with roles of IFN-λ in activation of antiviral defences of cells expressing corresponding receptors (73–75). In support, IFN-λ receptor KO mice showed higher RSV, human metapneumo- and influenza virus yields, and increased inflammatory responses compared to wild-type mice (76, 77). Neutrophils utilize three major protective mechanisms: phagocytosis, degranulation (emitting an array of anti-microbial substances, expression IFN stimulatory genes), and neutrophil extracellular traps (78). However, these defensive roles of neutrophils appear to be secondary due to their low numbers in lungs of OGG1 inhibitor-treated mice. Because the goal of this study was to elucidate the epigenetic role of 8-oxoGua in enhanced IFN-λ expression, its role(s) in anti-RSV infection still needs to be determined in future studies. The strategy of inhibiting OGG1 function by TH5487 will provide appropriate curtailment of the early response to prevent immune pathology (79).

In IFN expression, DNA cytosine methylation at CpG repeats are essential regulatory entities at sites where 8-oxoGua is generated. Oxidation of Gua to 8-oxoGua opposite to cytosine or in proximity (2-3 bases) inhibits the function of the DNA methyltransferases (DNMTs) (80, 81). Moreover, methyl binding proteins [MBP(s)] recognize Gua’s O6 and N7; however, oxidation of Gua converts the N7 from a hydrogen acceptor to a hydrogen donor and replaces the proton-8 with an oxygen atom, resulting in interference with methyl-CpG dinucleotide recognition by MBPs (82–84). To this end, the family of Ten-eleven translocation (TET) proteins are responsible for the enzymatic oxidation of 5mC into 5-hydroxymethylcytosine (5hmC), and 5-carboxylcytosine, which are substrates for thymidine DNA glycosylase and repaired via the BER pathway. The interference of 8-oxoGua function of MBPs imply that a group of genes may utilize OGG1 the reader of 8-oxoGua to override the repression by 5mC to execute a prompt cellular response to stimuli. While oxidative stress persists, OGG1 may further recruit TET1 for enzymatic catalysis of 5meC into 5hmC and thus the OGG1-initated pathway could be utilized to transfer the DNA oxidation signal to downstream of DNA demethylation enzymes (84). Gua oxidation is non-enzymatic thus, OGG1 complexed with its substrate might be the most economical strategy for cells to override repression by 5mC. The biological function of DNA methylation associated with 8-oxoGua generation in inflammatory processes and specifically with regulation of IFN-λ needs further characterization.

In this study, we found that RSV-infection generated oxidatively modified Gua lesions 8-oxoGua in promoters, and its reader, OGG1, impact antiviral gene expression. We propose that OGG1 by rotationally diffusing along the DNA helix, a mechanism facilitated by thermal activation locates its mark 8-oxoGua and may also influence chromatin remodelers. Also, it is yet to be discovered whether formation of the epigenetic mark 8-oxoGua and docking of the reader OGG1 precedes changes in chromatin architecture by histone modifications (such as acetylation, methylation, phosphorylation, and others) at the N- and C-terminal tails of histones (85). Given that the cellular redox state via posttranslational modifications affects OGG1 function, whether as a reader or eraser, both roles fit very well into the present hypothesis. We conclude that the epigenetic regulation is a highly dynamic process and, therefore, facilitates rapid phenotypic changes for the host. To this end, our work introduces a new concept –pharmacological regulation of OGG1 function as a reader and eraser to control the outcome of viral infections, which may also represent a general antimicrobial strategy in the future.

## Data availability statement

The original contributions presented in the study are included in the article/**Supplementary Material**. Further inquiries can be directed to the corresponding author.

## Ethics statement

The animal study was reviewed and approved by University of Texas Medical Branch (UTMB) Animal Care and Use Committee (approval no. 0807044D).

## Author contributions

YX, LP, XZ, KW, SV, and IB designed, performed, analysed cell culture and animal experiments. LP and IB wrote the manuscript. KW helped XZ to perform binding assays and immunoprecipitation. KW, LP, and XZ generated and characterized expression vectors. KW and YX generated OGG1 knock out hSAECs using CRISPR/Cas9 genome editing and characterized isolated clones. SV, LT, ZR, and ARB helped with writing, experimental designed, performed, some of the experiments. ARB and XB advised ChIP experiments. All authors discussed results and approved the content of the manuscript.
