# Supplementary material for: Epigenetic control of type III interferon expression by 8-oxoguanine and its reader 8-oxoguanine DNA glycosylase1
Source: Front Immunol. 2023 Aug 4;14:1161160. doi: 10.3389/fimmu.2023.1161160 (PMC10436556; doi:10.3389/fimmu.2023.1161160)
Supplement: Supplementary file 1 [file DataSheet_1.pdf]

Supplementary Material

Epigenetic Control of Type III Interferon Expression by 8-Oxoguanine and its Reader 8-Oxoguanine DNA Glycosylase1

Yaoyao Xue[1,2,#], Lang Pan[1,#], Spiros Vlahopoulos[3], Ke Wang[1,2], Xu Zheng[1,2], Zsolt Radak[4], Attila Bacsi[5], Lloyd Tanner[6], Allan R. Brasier[7], Xueqing Ba[2], Istvan Boldogh[1,*]

[1]Department of Microbiology and Immunology, University of Texas Medical Branch, Galveston, Texas 77555, USA

[2]Key Laboratory of Molecular Epigenetics of Ministry of Education, School of Life Science, Northeast Normal University, Changchun 130024, China

[3]Horemeio Research Laboratory, First Department of Pediatrics, National and Kapodistrian, University of Athens, 11527 Athens, Greece

[4]Research Institute of Molecular Exercise Science, University of Sport Science, H-1123 Budapest, Hungary

[5]Department of Immunology, Faculty of Medicine, University of Debrecen, 4032 Debrecen, Hungary

[6]Respiratory Medicine, Allergology & Palliative Medicine, Lund University and Skåne University Hospital, Lund, Sweden

[7]Department of Medicine, School of Medicine and Public Health, University of Wisconsin-Madison, Madison, WI 53705, USA

[#] **Equally contributed**

*** Correspondence:**
Istvan Boldogh, DM&B, PhD
sboldogh@utmb.edu.

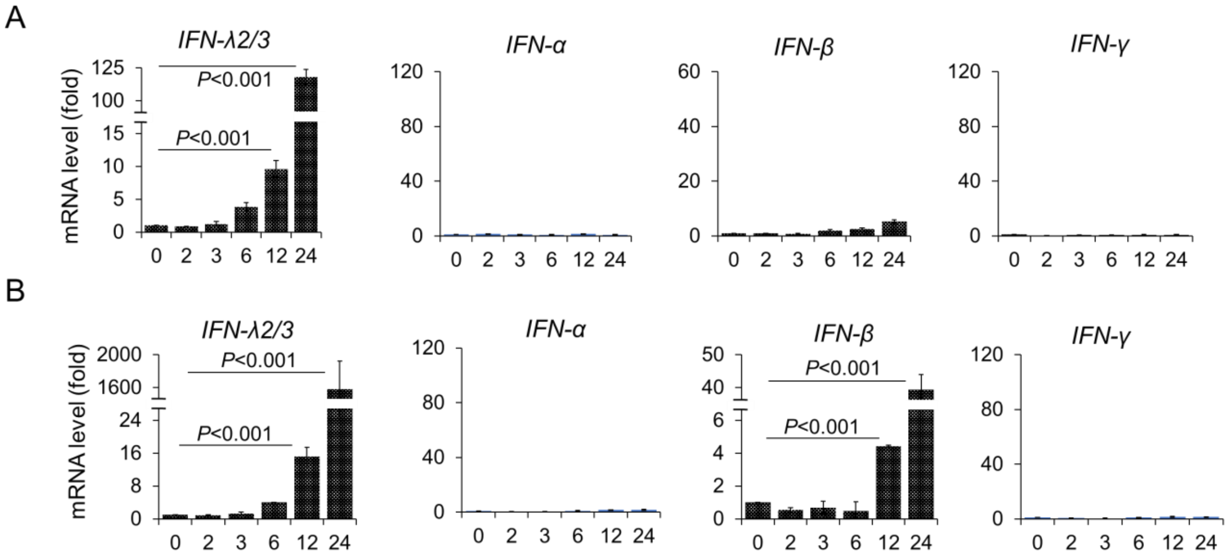

Supplementary Fig 1: Cell type specific differences in expression of IFNs after RSV infection.

(**A**) mRNA expression levels of IFNs in hSAECs after RSV infection. (**B**) mRNA expression levels of IFNs in A549 cells after RSV infection. In A and B, hSAECs or A549 cells at ~70% confluence were RSV-infected (MOI = 3), washed after 1h adsorption and new medium was added. Cells were harvested as indicated in the panels. Total RNA was isolated and after cDNA synthesis qPCRs were performed to determine mRNA levels of *INF-λ*2/3, *IFN-α*, *IFN-β* and *IFN-γ*. Data is representative of 3 independent experiments containing 2 biological replicates.

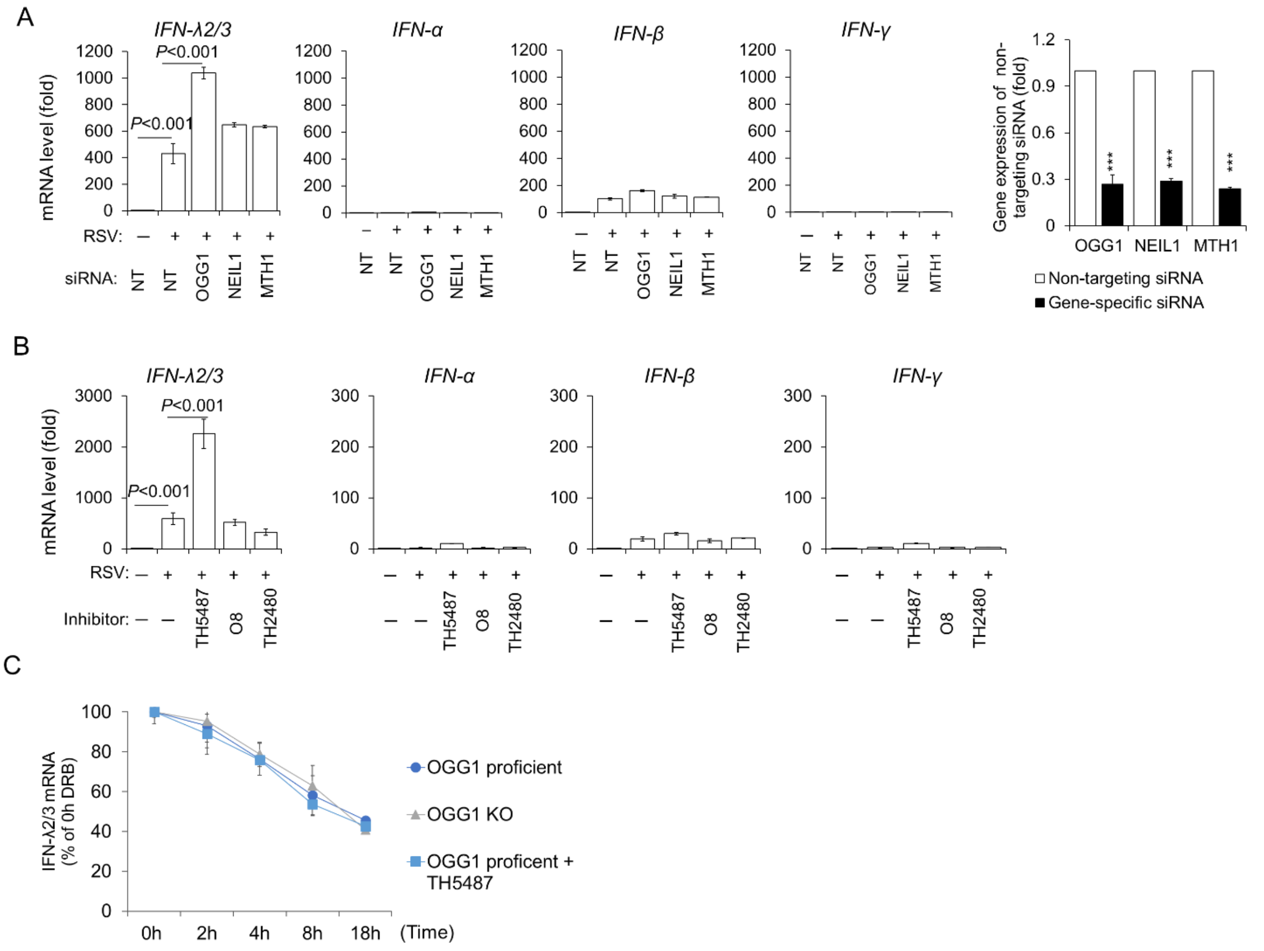

Supplementary Fig 2: OGG1 depleted cells, but not NEIL1, or MTH1 increased RSV infection induced IFN-λ2/3 expression.

(**A**) mRNA expression levels of *IFNs* are shown in A549 cells after RSV infection. Parallel cell cultures were transfected with target siRNA to deplete OGG1, Nei-like DNA glycosylase 1 (NEIL1) and MutT homolog 1 (MTH1) for 24 hours. Control cells were transfected with scrambled non-targeting siRNA (Materials and Methods). Depleted cell cultures were infected with RSV (MOI = 3) and harvested at 24 hpi. Total RNAs were isolated for assessment of mRNA depletion by qRT-PCR. (**B**) mRNA expression levels of *IFNs* are shown in A549 cells after RSV infection ± TH5487, O8 or TH2480. A549 cells at ~70% confluence were RSV-infected (MOI = 3), washed and TH5487 (10 µM), O8 (10 µM) or TH2480 (10 µM) were added. All inhibitors were added again at 12 hpi with change of media. Cells were harvested at 24 hpi. mRNA levels of *INF-λ2/3*, *IFN α*, *IFN-β* and *IFN-γ* were determined by qRT-PCR. (**C**) mRNA half-life of *IFN-λ*2/3 in OGG1 proficient and deficient cells. Cells were poly(I:C) treated for 30 min and monolayers were washed with PBS. Four hours later 20 µg/mL 5,6-dichloro-1-β-D-ribofuranosylbenzimidazole (DRB) was added. Total RNAs were isolated at DRB addition (0 h), 2, 4, 8 and 18 h as in Materials and Methods. The quantities *IFN-λ*2/3 mRNAs at each time points were determined by qPCR by normalizing to 18S rRNA. The relative amount of *IFN-λ*2/3 mRNA at time 0 h of DRB addition was set at 100% in each cell type (OGG1 proficient, OGG1 KO cells or hSEACs with functionally inactivated OGG1). Data is representative from 3 independent experiments. Statistical analysis, Student's t-tests (unpaired).

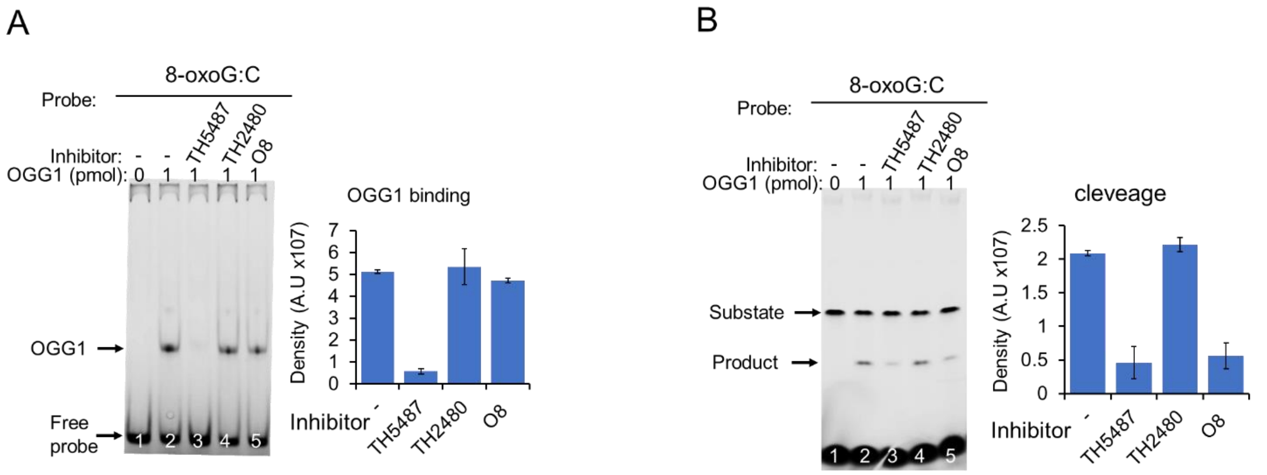

Supplementary Fig 3: TH5487 but not O8 or TH2480 inhibits OGG1 substrate binding and activity.

(**A**) Inhibition of OGG1 binding to its substrate by TH5487 as shown by the electrophoretic mobility shift assay (EMSA). Left panel: one pmol recombinant OGG1 (Item #: ENZ-253, ProSpec) ±inhibitor (10 µM) or vehicle was incubated with double-stranded DNA probe containing 8-oxoGua (see below) were mixed in buffer containing 10 mM Tris-HCl (pH 8.0), 10 mM NaCl, 1 mM DTT, 1 mM EDTA, 1 mg/ml BSA and 0.1 µg/µl poly(I:C) and incubated for 15 min on ice. Protein-DNA complexes were resolved on a 6% DNA retardation gel (Invitrogen, Item # EC6365BOX) in 0.5 × TBE buffer (100V for 2h) and bands were visualized by using the Amersham Imager 680 (Global Life Sci. Sol. Marlborough, MA). Right panel, band intensities were quantified using Image J v1.51 (U. S. NIH, Bethesda, Maryland, USA). (**B**) Inhibition of OGG1 DNA base excision activity by TH5487 and O8 but not TH2480 (an inactive analog of TH5487) using an oligonucleotide excision assay. In brief, 100 fmol of the Cy5 labeled probe (below) were incubated with 1 pmol of recombinant OGG1 (Item #: ENZ-253, ProSpec) in 10 µL digestion buffer (10 mM of Tris-HCl (pH 7.5), 10 mM of NaCl, 1 mM of EDTA, 1 mg/mL BSA, and 1 mM of DTT). After incubation for 10 min at room temperature, the reaction was stopped by adding 10 µL loading buffer (containing 8 µL of formamide, 10 mM of NaOH) and heated for 5 minutes at 95°C. The cleaved product was separated from the intact probe in a 15% polyacrylamide gel containing 8 M urea in Tris-borate-EDTA buffer (pH 8.4). The separated bands were visualized using the Amersham™ Imager 680 (Global Life Sci. Sol. Marlborough, MA). Right panel, band intensities were quantified using the Image J v1.51 (U. S. NIH, Bethesda, Maryland, USA).

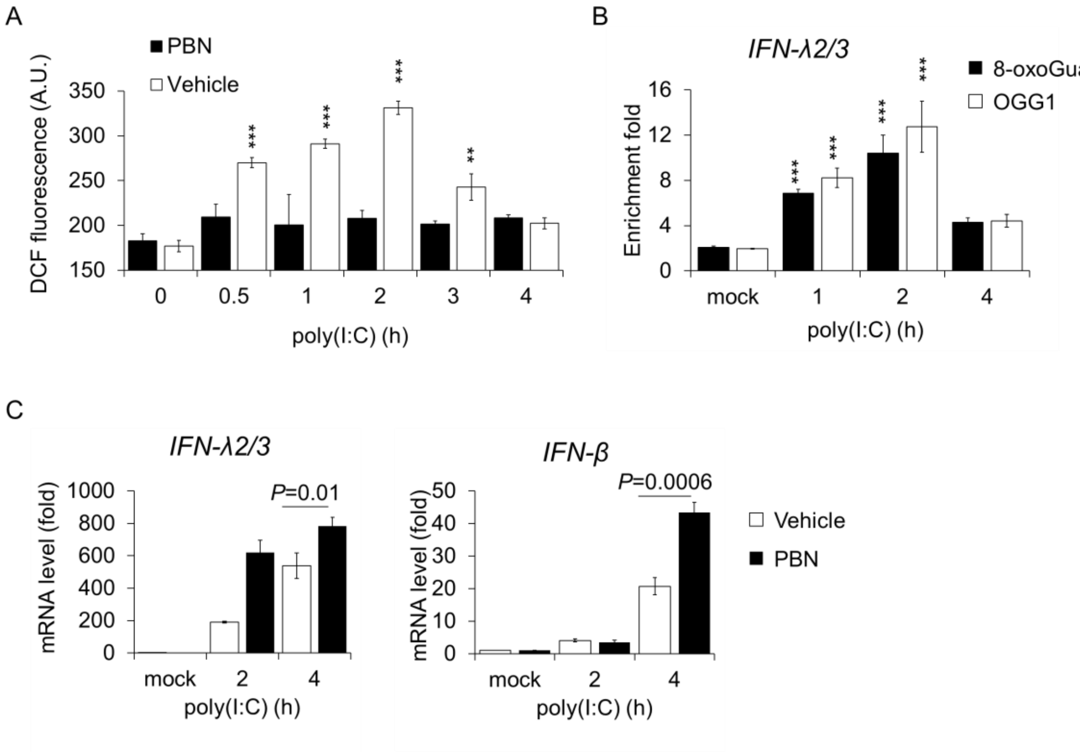

Supplementary Figure 4. Poly(I:C)-induced oxidative stress inversely correlates with mRNA levels of INF-λ2/3 and IFN-β.

(**A**) Parallel cultures of hSAEC were treated by poly(I:C) and/or phenyl-alpha-tert-butyl nitrone (PBN, 100 µM). Changes in ROS levels were determined by DCF assay (Materials and Methods). Data is representative of one experiment with 4 biological replicates. (**B**) Enrichment of 8-oxoGua and OGG1 on TSS adjacent promoter region of *IFN-λ* (for sequences see Supplementary Figure 5) after poly(I:C) (100 µg per mL) treatment. In brief, after 0, 1, 2, and 4 h postexposure with poly(I:C), 1×10^7 cells were cross-linked in 1% formaldehyde for 10 min followed by mixing with 1×Glycine for 5 min and chromatin immunoprecipitation was performed using specific antibodies and isotype control IgG. The DNA was phenol/chloroform-extracted, and levels of ChIP-ed DNA was determined by qRT-PCR performed in triplicate using SYBR Green PCR Master Mix (Bio-Rad) in a CFX 96 real-time PCR detection system (Bio-Rad) (Materials and Methods). Sequence of primers are listed in Supplementary Table 2. Data is representative of two experiments containing 2 biological replicates. (**C**) The antioxidant PBN increases poly(I:C) induced expression of *IFN-λ2/3* and *IFN-β*. mRNA levels were determined by qRT-PCR as in Materials and Methods. Data is representative of two experiments with 3 biological replicates. *p* values were calculated by two-tailed Student's t-tests (unpaired). $^{**}p < 0.01$, and $^{***}p < 0.001$

(h) IFN-λ2: -1000 to +100

ctcacgcctataatcccagaactttgggaggccgaagaaggcagatcacgaggtcaggagttcaagaccaacctggccaatat
ggcaaaaccccgtctctactaaaaatacaaaaattagttgggcgtggtggtgcatgcctatagtcctttctactcgggaggctgaa
gcagaagaatcccttgaaccaaggaggtggaggttgcagtgagtcgagatcacgctgctgcactccagcctgggctacagag
caagactccatctcaaaaaaaaaagaaaaaagaaagaaagaaagaaatcatggcctctgggcacagtggctcatgcctgcaa
ccccagcaatttgggaggccaagacagacagatcacttgacgtcaagagttcgagaccagcctggccaattggtgaactgtca
tctctactaaaaccataaaaattagctgggaatggtggcacaaatctgtaatctcagctacttgggaggctaaggcaagagaatc
gcttgaacccaggaggtggaggttgcagttagtcaagattttgcactgcactccagcctgggtgaccgaacaagaccctgtctc
aaaatatatatatatatatatgccaggaggggtggctcaggcctgtaatctcagcactttaataggctgggtgaggaggatggctt
gagcccaggagtttgaggctgcagtgagctgtgatcatgccattgcactccagtgacagagtgagaccctgtcttaaacaacaa
caaaaccagagcaggtggaatcctcctgggaacataccttcctgtaggttacccctgagtctccatcagtttctctttccctccagc
tgctcatctggctcactagccctgccctgctctgggctttcccagcctggggctcccctggtggccggtgtcttacctgaggctgt
gttttcacttttcctacatcagctgggactgcccttctgtcagggataaaagctgccccatggagctcaggcAGGAATTAC
ATCCCAGACAGAGCTCAAAACTGACAGAAAGAGTCAAAGCCAGGACACAGT
CTGAGATCCAGAAGAGGGGACTGAAAAGAACAGAGACTCC

(product: 188bp)
(h) IFN-λ2/3-F (-204/-17): CTGTAGGTTACCCCTGAGTC
(h) IFN-λ2/3-R (-204/-17): GCAGCTTTTATCCCTGAC

Supplementary Figure 5. Sequence of human IFN-λ promoter -1000 to +100 derived from Eukaryotic Promoter Data base (EPD)
https://epd.epfl.ch/search_EPDnew.php?query=interferon+2+promoter&db=human

Sequence and location of ChIP primers (Red fonts yellow highlighted). Sequence and position of IRF binding site (black characters yellow highlighted). Sequence and location of NF-κB/p50-p65 binding site (black fonts, blue highlights). Potential binding sites for NF-κB1/P50-p50 (5’-GGG-3’) in sense and antisense strands are shaded gray. Size of ChIP-ed DNA was 183 bp. The underlined sequence was used as a probe in gel shift and binding assays.
The sequence of the wild-type probe used in EMSA.
5’-CTGTGTTTTCACTTTCCTACATCAGCTGGGACTGCCCTTCTGTCAGGGATAA-3’
For the mutated probes please see Table 1.

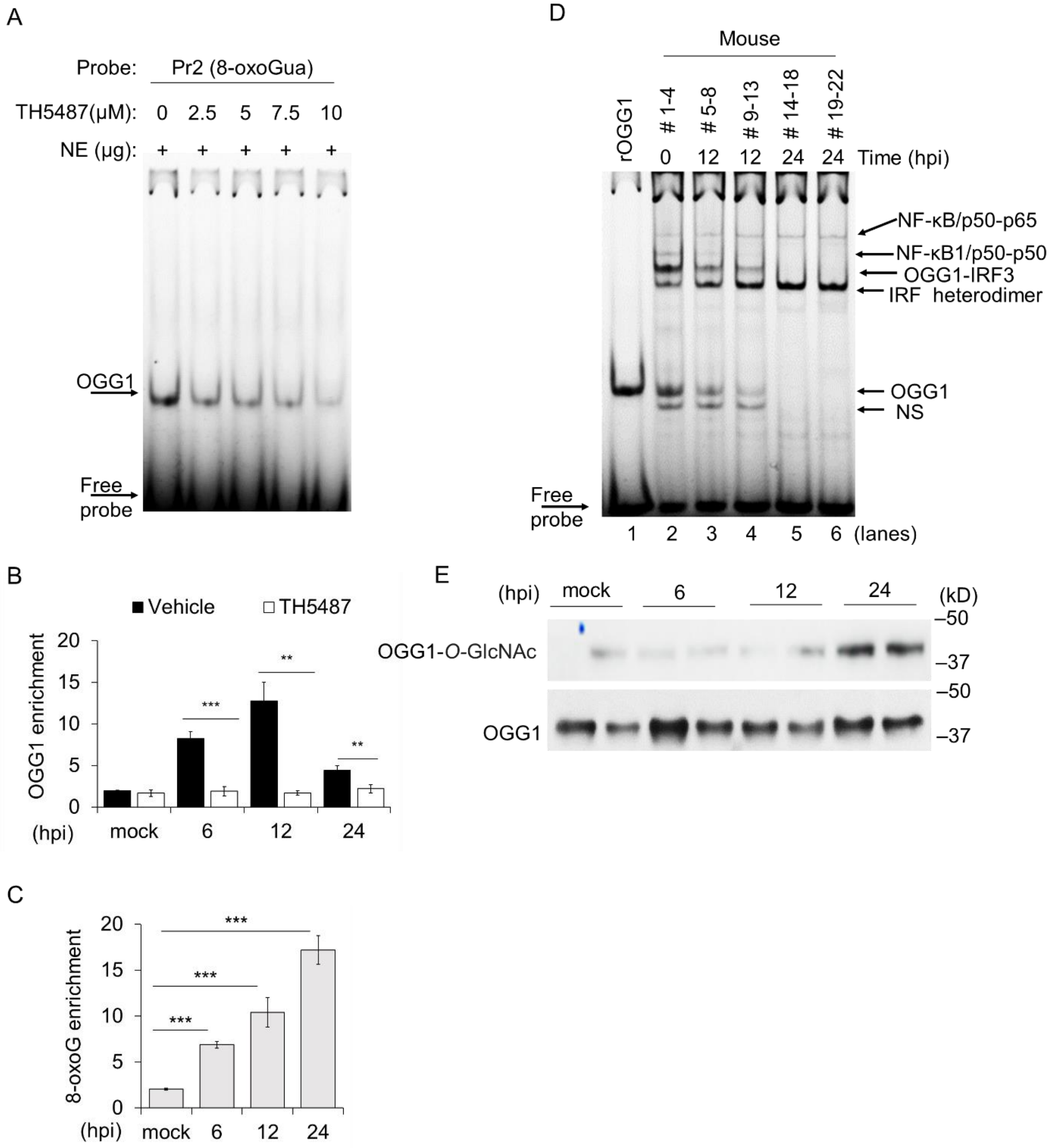

Supplementary Figure 6. **RSV induced OGG1 modification and recruitment to *IFN-λ2/3* promoter in mouse lungs.**

(**A**) Inhibition of OGG1 binding to its substrate by TH5487 as shown by the electrophoretic mobility shift assay (EMSA). Probe sequence is shown in Table 1. (**B**) OGG1 binding to the *IFN-λ2*/3 promoter. Mice were treated with TH5487 or vehicle and RSV infected (10^6 per lung). ChIP assays were carried out using antibody to OGG1. *p* values were calculated by two-tailed Student's t-tests (unpaired). $**p < 0.01$, and $***p < 0.001$. (**C**) 8-oxoGua levels in the promoter of *IFN-λ*2/3 as shown by ChIP assays. DNA were extracted from lungs of RSV (10^6 per lung)-infected mice, and immunoprecipitation (IP) was carried out with 8-oxoGua antibody. The level of DNA was quantified

by qRT-PCR. *p* values were calculated by two-tailed Student's t-tests (unpaired). $^{**}p < 0.01$, and $^{***}p < 0.001$. (**D**) EMSA showing binding of NF-κB, IRF3 and OGG1 to the Pr2 probe (Table 1). Mice were mock- or RSV-infected for indicated time, and NEs were prepared from lungs. (**E**) Western blot shows the O-linked N-glycation levels of OGG1. Mice were mock- or RSV-infected for indicated time, and lungs lysates were prepared. Immunoprecipitants by OGG1 antibody were subjected to PAGE and immunoblotting was performed using OGG1 and O-GlcNAc (RL2) antibodies. Upper panel, OGG1-O-GlcNAc, Lower panel, unmodified OGG1.

Supplementary Table 1: Primer sequences for qRT-PCR

Gapdh	F: 5′-AATGGTGAAGGTCGGTGTG-3′<br>R: 5′-GTGGAGTCATACTGGAACATGTAG-3′
Ifn-α	F: 5′- ATGGCTAGGCTCTGTGCTTTCC<br>R: 5′-TCCTCACAGCCAGCAGGGAGT
Ifn-β	F: 5′-CAGCTCCAAGAAAGGACGAAC<br>R: 5′-GGCAGTGTAACTCTTCTGCAT
Ifn-γ	F: 5′-GCTTTGCAGCTCTTCCTCA<br>R: 5′-TTTCTTCCACATCTATGCCACT
Ifn-λ2/3	F: 5′-TCCTCCTGCTGTTGCCTCTG<br>R: 5′-GGTGGGAACTGCACCTCA
Mx1	F: 5′-TCTGAGGAGAGCCAGACGAT-3’;<br>R: 5′-ACTCTGGTCCCCAATGACAG-3’
Isg15	F: 5′-GCTCCAGGACGGTCTTAC<br>R: 5′-TGGTCTTCGTGGACTTGTT
Oas1a	F: 5′-TGTCAAATCAGCCGTCAA<br>R: 5′-AGGAACACCACCAGGTCA
Rsad2	F: 5′-CCCTCTGTGAGCATAGTGA<br>R: 5′-AGCCACCTTGTAATCCCT
GAPDH	F: 5′-ACATCGCTC AGACACCATG-3′<br>R: 5′-TGTAGTTGAGGTCAATGAAGG G-3′
IFN-α	F: 5′-CCCATTTCAACCAGTCTAGCAG<br>R: 5′-TGTGGGTTTGAGGCAGATC
IFN-β	F: 5′-TGCTCTGGCACAACAGGTAG<br>R: 5′-CAGGAGAGCAATTTGGAGGA
IFN-γ	F: 5′-CCAACGCAAAGCAATACAAGA<br>R: 5′-TTTTCGCTTCCCTGTTTTAGC
IFN-λ2/3	F: 5′- AGTGCTGACCGTGACTGGA<br>R: 5′-GCGACTCTTCTAAGGCATCTT
TNF	F: 5′-TGCACTTTGGAGTGATCGG-3′<br>R: 5′-TCAGCTTGAGGGTTTGCTAC-3′
OGG1	F: 5′-CATATGAGGAGGCCCACAAG-3′<br>R: 5′-CAGAAGATAAGAGGACGCAGAAG-3′

NEIL1	F: 5′- GACAGAGGCAAGTGGCAA AGCA-3′<br>R: 5′- GCCTCATTCACAAACTGGCTGG-3′
MTH1	F: 5’-GTCTTCTGCACAGACAGCATCC-3’<br>R: 5′-CTGAAGCAGGAGTGGAAACCAG-3’

Supplementary Table 2: Primer sequences for ChIP-coupled qRT-PCR

IFN-α (-184/-4)	F: 5’-AAGGCTCTGGGGTAAAAGA-3′<br>R: 5′-GACCTTGCTTTGTGCCTAGC-3′
IFN-β (-150/+5)	F: 5’-TCATAAGATAGGAGCTTAAA-3′<br>R: 5′-GAATGTCCTTTCTCCATGG-3′
IFN-γ (-142/+54)	F: 5’-AATGCCACAAAACCTTAGTTATTAA-3′<br>R: 5′-ACTTAACTGATCTTTCTCTTCTAAT-3′
IFN-λ (-204/-17)	F: 5’-CTGTAGGTTACCCCTGAGTC-3′<br>R: 5′-GCAGCTTTTATCCCTGAC-3′
TNF (-350/-120)	F: 5′-GGTCCCCAAAAGAAATGGAGG-3′<br>R: 5′-TTTATATGTCCCTGGGGCGA-3′
IL6 (-267/-117)	F:5-GCAGATGAGTACAAAAGTCCTGA-3′<br>R: 5′-TTCTGTGCCTGCAGCTTC-3′
CXCL10 (-179/+71)	F: 5′-AGGGAA ATTCCGTAACTTGGA GGC-3’<br>R: 5′-TTCATGGTGCTGAGACTGGAGGTT-3’

REFERENCES:

Zheng, X., Wang, K., Pan, L., Hao, W., Xue, Y., Bacsi, A., Vlahopoulos, S. A., Radak, Z., Hazra, T. K., Brasier, A. R., Tanner, L., Ba, X. and Boldogh, I. (2022). "Innate Immune Responses to RSV Infection Facilitated by OGG1, an Enzyme Repairing Oxidatively Modified DNA Base Lesions." J Innate Immune 14(6): 593-614.
